# A fuzzy set qualitative comparative analysis of 131 countries: which configuration of the structural conditions can explain health better?

**DOI:** 10.1186/s12939-018-0724-1

**Published:** 2018-01-22

**Authors:** Toktam Paykani, Hassan Rafiey, Homeira Sajjadi

**Affiliations:** 1 Department of Social Welfare, School of Education Sciences, University of Social Welfare and Rehabilitation Sciences, Tehran, Iran; 2 Social Welfare Management Research Center, University of Social Welfare and Rehabilitation Sciences, Tehran, Iran

## Abstract

**Background:**

According to the recommendations of the World Health Organization Commission On Social Determinants of Health (CSDH) for intersectoral action on health, the well-being of and equity in health within a population are achieved via a complex fusion of policies and actions. In this study, following the CSDH’s approach and considering set-theoretic relations, we aimed to unravel this complexity and answer the kinds of questions that are outside the scope of conventional variable-oriented approach.

**Methods:**

A fuzzy-set qualitative comparative analysis of 131 countries was conducted to examine the configurational effects of five macro-level structural conditions on life expectancy at birth. The potential causal conditions were level of country wealth, income inequality, quality of governance, education, and health system. The data collected from different international data sources were recorded during 2004–2015.

**Results:**

The intermediate solution of the truth table analysis indicated a configuration of conditions including high level of governance, education, wealth, and affluent health system to be consistently sufficient for high life expectancy. On the other hand, four configurations, each containing two or three conditions, were consistent with being usually sufficient to cause low life expectancy.

**Conclusions:**

We were able to configurationally explore the cases and specify the combinations of potentially causal conditions which were usually sufficient to explain high or low life expectancy in different countries. As a result, particular cases were identified for further research. In addition, research may provide **s**upport for the CSDH’s recommendations emphasizing the importance of intersectoral action for health.

**Electronic supplementary material:**

The online version of this article (10.1186/s12939-018-0724-1) contains supplementary material, which is available to authorized users.

## Background

Today, there is a 34-year gap between countries with the highest and lowest life expectancies [38]. However, no biological explanation has not been found for such a difference [18, 41].

It is now generally accepted that inequalities root in the conditions under which people are born, grow up, live, work, and grow older. Such conditions of daily life, also referred to as social determinants of health (SDH), are linked to several systems and forces, including political systems, social policies, economic policies, and social norms [18, 29]. In 2005, the World Health Organization (WHO) established the Commission on Social Determinants of Health (CSDH) to attract the attention of the governments and civil society to the effects of social factors upon health. Under the broad category of health determinants, The CSDH categorizes health determinants into structural determinants (those leading to health inequalities), intermediary health determinants (including material, psychosocial, behavioral, and/or biological factors), and the health system itself as a social determinant [29]. The CSDH’s final report provided a large body of evidence on what can be done to reduce health inequalities within and between countries. The commission made it perfectly clear that policies for health equity involve a wide range of sectors with different core activities.

**Fig. 1:**
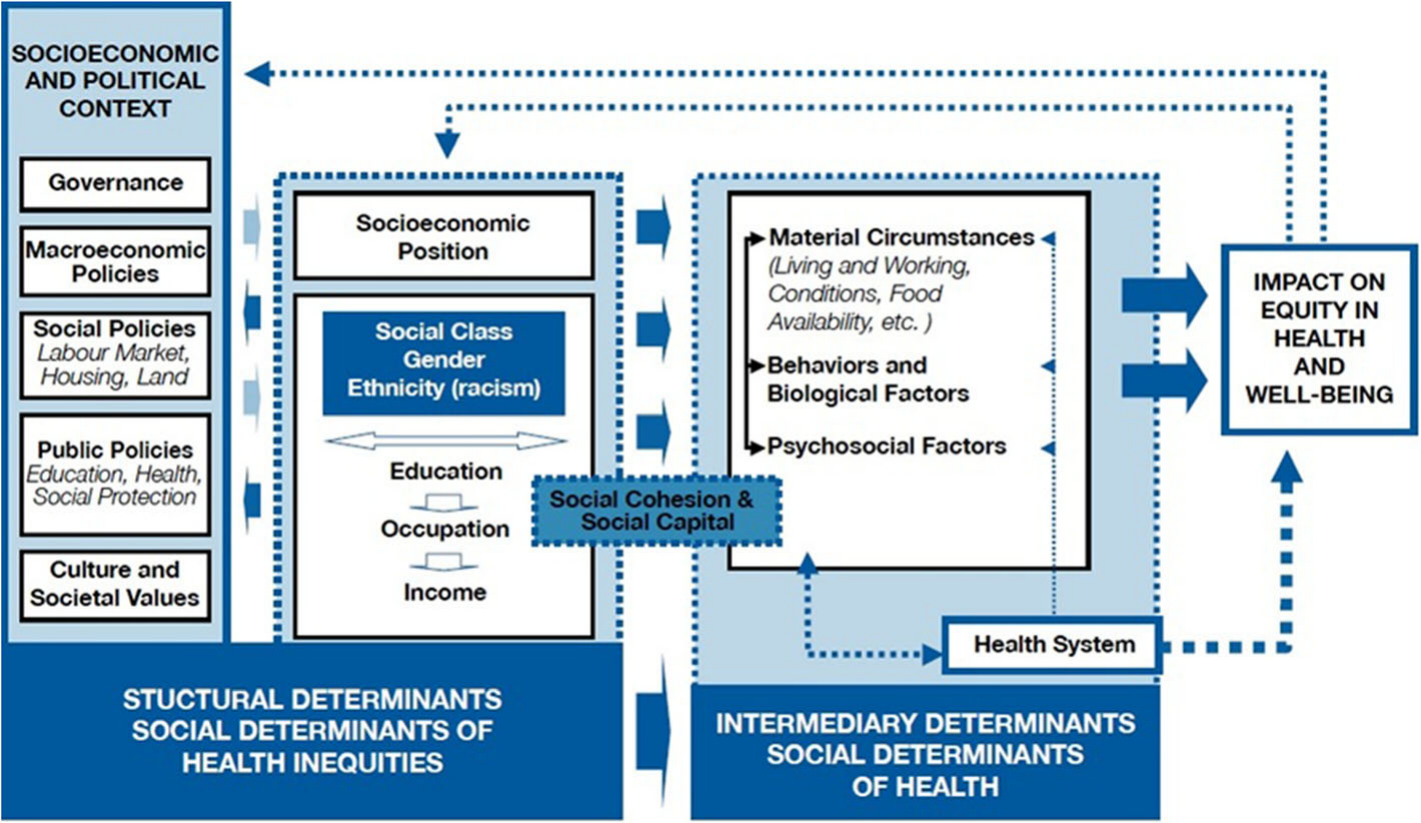
Final form of the CSDH conceptual framework brings together the key elements -including structural and intermediary determinants- and the processes and pathways that generate health inequities [29]

Figure 1 depicts the conceptual framework of the CSDH with all its elements. As seen, the interactions between various socioeconomic and political factors, such as gender, race/ethnicity, income, education, occupation, and other factors, form a set of socioeconomic positions which affect the intermediary determinants of health. These determinants, which reflect a person’s position in the social order, are influenced by one’s social status and experiences of exposure and vulnerability to health-compromising conditions. Overall, The CSDH has subcategorized health determinants as the sociopolitical context, structural determinants and socioeconomic position, and intermediary determinants [29].

It should be noted that this framework is distinguished from previous ones by its emphasis on the socioeconomic and political context and the structural determinants of health inequality. Therefore, the most important lesson learned by the CSDH during the dialogue between idea and evidences on social determinants of health was that in order to minimize health inequalities, governments should focus on not only intermediary determinants of health, but also the underlying structural determinants [17, 18, 29]. Since policies aiming at structural determinants of health inequalities require the involvement of main institutes distributing power and resources within the society, they should be carefully designed based on methodologies developed by social and political science and according to contextual specifications [18, 29].

Since the CSDH published its conceptual framework for tackling the social causes of health inequalities, extensive research has been conducted on the empirical relevance of the socioeconomic variables discussed in the CSDH report [1, 7, 10, 14, 42]. Such quantitative research assessed the relationship between socioeconomic disparities and health gradients and the degree to which social factors could explain health inequalities. The considerable evidence compiled by these studies identified the “net effect” of particular socioeconomic variables on health outcomes [20].

Nonetheless, the idea that “complexity defines health” is the core focus of the policies recommended by the CSDH [17, 29]. This notion implies that the production of positive health outcomes requires interaction and coordination between the health sector and other non-health-related sectors. Indeed, the CSDH conceptual framework presents a complex and nonlinear causal model of health equity. Accordingly, this study aimed to unravel these causal complexities by developing a fuzzy set Qualitative Comparative Analysis (fsQCA) [19, 20].

The outcome of interest for this internationally comparative study was life expectancy at birth. This focus was selected due to the status of life expectancy as the most general indicator of health outcomes and a main concern in health-related Sustainable Development Goals [29, 38]. Life expectancy at birth is an estimate of the average number of years that a newborn is expected to live if current mortality rates continue to apply. It indicates the overall mortality level of a population [36]. A large body of literature has shown the relationship between socioeconomic conditions and life expectancy, i.e. socioeconomic health disparities have been found to be reflected in life expectancy [2, 4, 7, 40, 42].

After an overview of all recommended policies discussed in the CSDH reports, some preliminary analysis^1^ was conducted and the authors identified five conditions as the most theoretically and empirically important drivers of socioeconomic and political conditions within the macro-level context in the CSDH framework. These conditions included [1] economic development (country wealth), [2] education, [3] quality of governance, [4] income inequality, and [5] health systems as an intermediary factor [29]. Furthermore, data for these five indicators are available at the national level with reasonable amount of missing value.

We applied fsQCA in order to assess the configurational effects of these five macro-level socioeconomic conditions. Our reasons for selecting fsQCA were related to the CSDH’s approach which emphasizes the need for multiple, intersectoral policies and actions on health. The major advantage of utilizing fsQCA for our study was that it enabled us to deal with causal complexity [20]. Moreover, the use of fsQCA facilitated the evaluation of cases as configurations of conditions, rather than the net effect of each variable on outcomes. We were able to conduct context-specific assessments indicating the ways in which multiple causal recipes linked to a particular outcome. Another advantage of using fsQCA in this study was its compatibility with macro-level data analysis in small- or medium*-*N research [20]*.* In fact, using this technique, we could answer certain questions that could not be approached via conventional variable-oriented analysis [19, 20]. More precisely, following the CSDH’s recommendations and considering set-theoretic relations, we were concerned with two main questions:

1. What combination of structural conditions is usually sufficient for high life expectancy?
2. What combination of structural conditions is usually sufficient for low life expectancy?

## Methods

### Data and measurements

#### Outcome

Our target research outcome was to identify factors related to life expectancy at birth. The required data on life expectancy were taken from the World Health Statistics Report 2017 [38].

#### Conditions

##### Governance

This condition was measured using the Worldwide Governance Indicator (WGI) developed by the World Bank [32]. The WGI assesses the quality of governance by examining six dimensions including [1] voice and accountability, [2] political stability and lack of violence, [3] government effectiveness, [4] regulatory quality, [5] the rule of law, and [6] control of corruption [11, 32]. We calculated the mean scores of each dimension for all included countries between 2004 and 2015. The average of the six dimensions was calculated as the overall score of governance for each country.

##### Wealth

The World Bank provides income data on gross domestic product (GDP) per capita adjusted for purchasing power parity (PPP) on an annual basis for about 232 countries [33]. The [8] defined GDP per capita as a country’s final product of total goods and service at market value divided by its population (both the residents and foreign labor) during a specific period of time. PPP determines a rate of exchange for $US to allow comparisons between countries (United Nations, [8]). For each country, the average GDP per capita was calculated for years 2005 to 2015.

##### Income inequality

Data on income inequality were extracted from the United Nations’ Human Development Index (HDI), 2015. However, finding a valid measure for income inequality was challenging. While the most commonly used measure of income inequality is the Gini coefficient [10], the Atkinson measure was used in this study due to (i) subgroup consistency (according to which overall inequality decreases if inequality decreases in one subgroup and stays the same in the remaining population), (ii) sensitivity to inequality in the lower range of distribution (the Gini coefficient places equal weights on the whole distribution, while the Atkinson inequality places greater weight on the lower end of the range and is thus a better measure for child mortality, illiteracy, and income poverty), and (iii) coverage of more countries (United Nations, [9, 30]). While the most commonly used measure of income inequality is the Gini coefficient Data on Atkinson measure of income inequality under the HDI dataset is available for years 2010 to 2015. Therefore, an average score for income inequality between years 2010 and 2015 was used for each country. The higher values of the Atkinson measure indicate greater inequality.

##### Education

The fifth pillar of the GCI, which is a composite indicator of the quality and quantity of secondary education, tertiary education, and job-training services, was applied [27]. The average GCI fifth-pillar score was determined for the years 2005–2015.

##### Health systems

We constructed a composite indicator of the health system strength. It included three sub-indexes [37]: (i) the workforce (numbers of physicians, nurses, and midwives per 1000 people), (ii) infrastructure (numbers of hospital beds per 1000 people), and (iii) health expenditure per capita. The information for all three sub-indexes was taken from the World Development Indicators of the World Bank’s database (2004–2014). First, all the raw values were standardized. Then, sub-index scores for the workforce were calculated by determining the means of standardized scores for physicians, nurses, and midwives. The overall scores for health systems were obtained by calculating the averages of the standardized scores for the three sub-indexes in each case.

We avoided handling missing data since fsQCA requires an in-depth examination of each case [20]. In fact, one’s confidence in any additional data would have to be very high to avoid misinterpretation of the information. Consequently, when using fsQCA, cases of missing data are removed from the study sample. Therefore, the analysis included 131 countries with no missing values (see Additional file 1).

### Analysis strategy

#### Step 1: The definition and labeling of the fuzzy sets

The most important step within fsQCA is the construction and calibration of the fuzzy sets. Indeed, the success of any fuzzy-set analysis depends on this step [20]. Due to the asymmetry in set-theoretic analysis, we defined two fuzzy sets [20, 26, 30], i.e. countries with high life expectancy and countries with low life expectancy, for the outcome. For each condition, a fuzzy set was defined and the degree of membership of each case in each of the fuzzy sets was determined. The fuzzy set membership scores ranged between 0 and 1 and indicated whether the cases had a high or low level of each condition [19].

#### Step 2: Calibration

In order to transform our interval-scale measures (see Additional file 2) into fuzzy sets, we used a direct method of calibration. The most important task in direct calibration is specifying the three qualitative anchors that structure a fuzzy set [20]. These anchors included [1] the threshold for full membership (fuzzy score = 0.95), [2] the threshold for full non-membership (fuzzy score = 0.05), and [3] the cross-over point or maximum ambiguity point (fuzzy score = 0.5). These three thresholds must definitely be based on theoretical and substantive knowledge of the cases [20]. Since this study dealt with more than 130 countries in a varying setting without definitive standards for areas of measurement, the threshold values were determined by the existing distribution and knowledge of the cases [6, 30, 31].

**Fig. 2:**
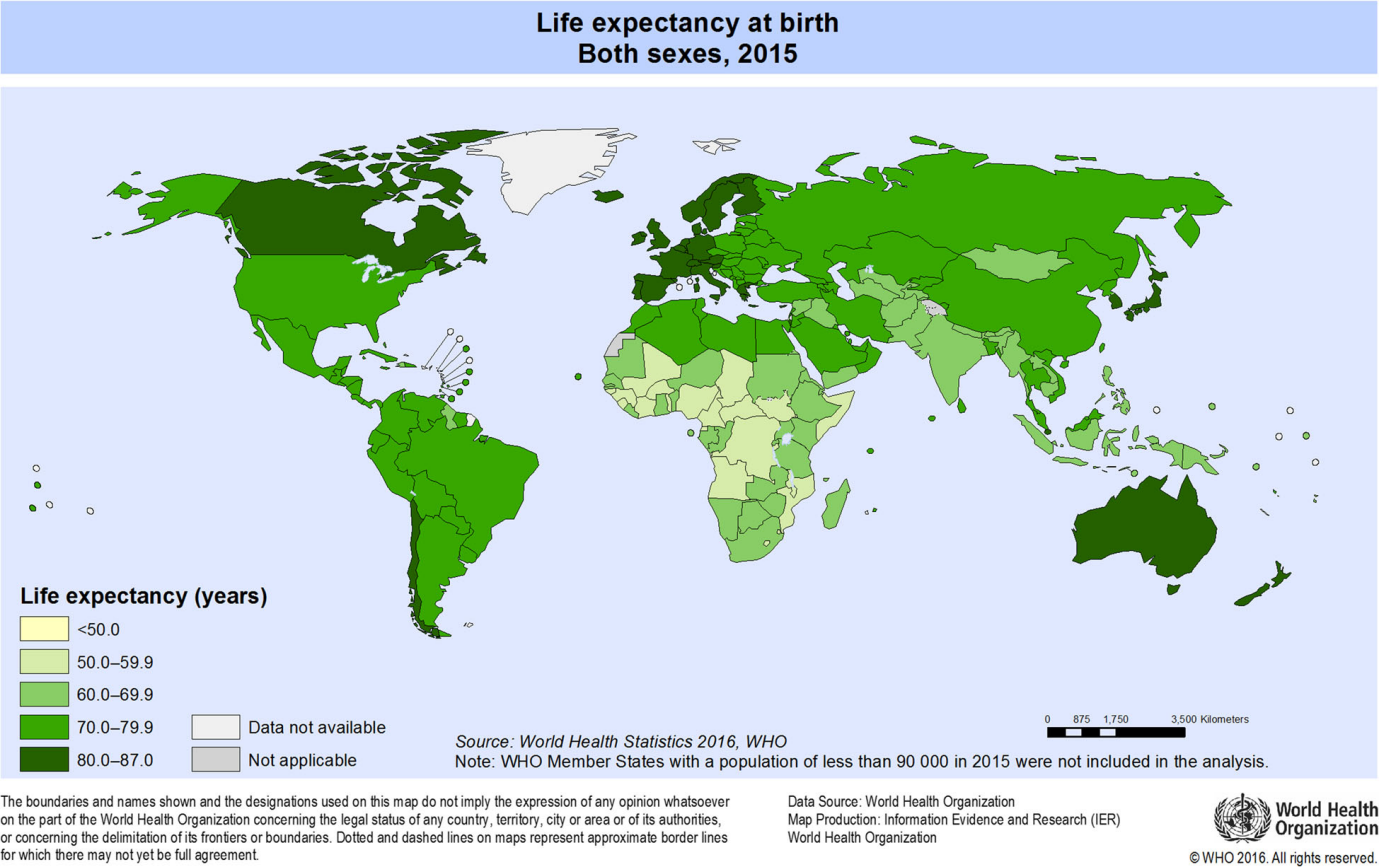
Life expectancy at birth, both sexes, 2015 (World Health Organization, 2016)

To calibrate the fuzzy sets for the relatively high level of each condition, the full membership threshold was set near the 80th percentile, the full non-membership threshold was set near the 20th percentile, and the cross-over point was set at about the 50th percentile on a value with no observation (see Additional file 3). To construct the fuzzy-set measures of countries with high and low life expectancy, substantive knowledge of the cases was derived from the WHO database (Table 1). The WHO grouped countries into five categories based on their life expectancy score (see Fig. 2). In this study, countries in category V (life expectancy scores > 80 years) were qualified cases with strong membership in the set of countries with high life expectancy. Countries in categories I (life expectancy score < 50 years), II (life expectancy score: 50–59.9 years), and III (life expectancy score: 60–69 years) were considered as countries with strong membership in the set of countries with low life expectancy. Table 2 shows the condition sets, the threshold value of the raw data above which cases were calibrated as fully included in the set of countries with a high level of that condition (fuzzy set score = 0.95), the crossover point of maximum ambiguity determining whether a case is more in or more out of a given set (fuzzy score = 0.5), and the threshold value below which cases were calibrated as being fully out of the set of countries with a high level of that condition (score = 0.05). Descriptive statistics of raw data areshown in Table 1.

**Table 1:** Descriptive statistics of data

Variable	Max	Median	Min
Life Expectancy at Birth	83.7	74.4	50.1
Education	6.11	3.98	2.01
Governance	1.85	−0.25	−1.46
Health system	2.5	−0.25	−0.91
Wealth	92,322	9912	714
Income Inequality	68.3	21.18	5.36

**Table 2:** Threshold values for calibration

Condition-set	Fully in	Crossover point (maximum ambiguity)	Fully out
High Life expectancy	80	75.2	68
Low Life expectancy	68	75.2	80
High quality education	4.86	4.09	3.05
Good governance	0.86	−0.21	−0.69
Affluent health system	0.9	−0.08	−0.7
High Income inequality	33	21.25	15
High wealth	28,500	10,500	3000

It should be noted that scores for the opposite pole of each condition (e.g. the degree of membership in a set of cases with a low, instead of high, level of a specific condition) can be calculated by using the principle of ‘set negation’ and subtracting the fuzzy-set scores from 1 [21, 30].

#### Step 3: Test of necessity

Based on set-theoretic relationships, if the fuzzy-set scores of a condition or configuration are consistently equal to or higher than the fuzzy-set scores for the outcome, a relation of necessity is indicated. It means that a causal condition is necessary for an outcome when instances of the outcome constitute a subset of instances of the causal condition [20]. As recommended by Ragin, we performed tests of necessity before conducting a sufficiency test [19, 26].

#### Step 4: Test of sufficiency

If the fuzzy-set scores of a condition or configuration are consistently equal to or smaller than their fuzzy-set membership scores on the outcome, this means that the condition or configuration is a fuzzy subset of the outcome. Such a set-relationship suggests that the condition is sufficient for the outcome [20].

A key tool for systematic analysis of causal complexity provided by fsQCA is the truth table [20]. The truth table is a tool that looks for configurations sufficient for the outcome. It lists all logically possible combinations of causal conditions, along with the empirical outcome connected to each configuration [20, 21]. This approach facilitates the analysis of causal complexity and allows for focused comparisons based on the empirical importance of different combinations of conditions [20].

The fsQCA software package applies minimization rules to simplify the Boolean expression (configurations). The truth table tool produces three solutions: [1] a complex solution that avoids using any remainders (i.e. logically possible configurations that lack empirical instances) in the minimization process; [2] a parsimonious solution which allows the incorporation of any remainder that will help generate a logically simpler solution regardless of their empirical plausibility and the existing substantive knowledge; and [3] an intermediate solution which permits the incorporation of only remainders that are expected to affect the outcome on the basis of previous empirical research [21]. Therefore, the existing knowledge is incorporated into the production of the intermediate solution [20, 30].

In this study, we conducted two fuzzy-set truth table analyses to identify the combinations of conditions that were consistently sufficient to cause the outcomes of either high or low life expectancy. Fuzzy set calibration and data analysis were carried out using the fsQCA software version 2.5 [22].

## Results

First, the positive and negative forms of five conditions were included in the necessity analysis. Based on the existing recommendations, the minimum acceptable level of consistency for the test of necessity was set at 0.965 [31, 34]. Accordingly, no individual conditions were found necessary for high or low level of life expectancy (Table 3).

**Table 3:** Analysis of necessary conditions

Conditions tested	High life expectancy		Low life expectancy	
	Consistency	Coverage	Consistency	Coverage
Education				
High	0.885	0.8	0.338	0.401
low	0.338	0.279	0.831	0.905
Governance				
High	0.857	0.760	0.356	0.416
low	0.341	0.287	0.794	0.88
Health system				
High	0.789	0.756	0.323	0.408
low	0.382	0.300	0.806	0.834
Income inequality				
High	0.433	0.382	0.635	0.738
low	0.703	0.594	0.468	0.521
Wealth				
High	0.897	0.799	0.351	0.411
low	0.339	0.284	0.828	0.914

### What combination of structural conditions is usually sufficient for high life expectancy?

With the five conditions, the truth table would have 32 (i.e. 2^5^) logically possible combinations of causal conditions. Table 4 displays the 12 combinations that had at least two cases with greater than 0.5 membership in the configuration. The minimum acceptable consistency for the solutions was set at 0.9. A consistency value above 0.9 indicated that the cases in the given configuration could be considered as strong subsets of the outcome [20]. Two configurations with consistency scores greater than 0.9 were considered as the subsets of the set of countries with high life expectancy (see rows 1 and 2 of Table 4).

**Table 4:** Distribution of cases across combinations of causal conditions

						Consistency for		
High Education	Good Governance	Affluent Health System	High Income Inequality	High wealth	number	high life expectancy	low life expectancy	Cases
1	1	1	0	1	32	**0.95**	0.222	Australia Austria Barbados Belgium Canada Cyprus Czech Republic Denmark Estonia Finland France Germany Greece Hungary Iceland Ireland Italy Japan Korea, Rep. Luxembourg Malta Montenegro Netherlands Norway Poland Portugal Slovak Republic Slovenia Spain Sweden Switzerland United Kingdom
1	1	1	1	1	4	**0.908**	0.564	Croatia Israel United States Uruguay
1	0	1	1	1	2	0.845	0.873	Argentina Lebanon
1	0	0	1	1	2	0.81	*0.964*	Colombia Thailand
1	1	0	1	1	4	0.791	0.785	Chile Costa Rica Malaysia Turkey
1	0	1	0	1	2	0.698	*0.946*	Kazakhstan Russian Federation
0	0	0	1	1	3	0.658	*0.984*	Dominican Republic Gabon Venezuela
0	1	0	1	1	4	0.631	*0.967*	Botswana Suriname Trinidad and Tobago South Africa
0	0	1	0	0	4	0.544	*0.988*	Armenia Kyrgyz Republic Moldova Tajikistan
0	1	0	1	0	6	0.429	*0.979*	Belize Bhutan Cape Verde El Salvador Ghana Namibia
0	0	0	0	0	14	0.282	*0.997*	Burundi Cambodia Cameroon Egypt Ethiopia India Lao PDR Liberia Mali Pakistan Sri Lanka Tanzania Timor-Leste Yemen
0	0	0	1	0	28	0.241	*0.97*	Angola Benin Burkina Faso Bangladesh Bolivia Côte d’Ivoire Guinea Gambia, The Guatemala Guyana Honduras Kenya Morocco Madagascar Mozambique Mauritania Malawi Nigeria Nicaragua Nepal Paraguay Rwanda Senegal Sierra Leone Swaziland Chad Zambia Zimbabwe

bold text indicates configurations that are sufficient for the outcomes of high life expectancy. Italic text indicates configurations that are sufficient for the outcomes of low life expectancy

It should be noted that there are two types of measurement for evaluating the strength of set relations: 1) set-theoretic consistency and 2) set-theoretic coverage. Set-theoretic consistency measures the level of agreement between cases sharing a particular combination of conditions in describing the target outcome. In other words, it determines the accuracy of the approximation of the perfect subset relation [20, 21]. On the other hand, set-theoretic coverage, measure empirical relevance/importance, i.e. it evaluates the level to which a cause or causal combination “accounts for” instances of an outcome. A given causal combination might have a small coverage in the presence of several paths to a specific outcome [20, 21]. In cases of equifinality, i.e. in the presence of two or more sufficient conditions or combination of conditions for an outcome, the coverage of alternate causal combinations is evaluated to obtain direct evidence of their relative empirical significance [20]. The fsQCA gives three coverage measures including solution, raw, and unique coverage which measure the proportion of memberships in the outcome that is explained by respectively the complete solution, each term of the solution, and each individual solution term (memberships that are not covered by other solution terms) [21].

Using fuzzy-set truth table analysis, the two configurations with consistency scores greater than 0.9 were summarized. As a result, one combination of causally relevant conditions linked to high life expectancy (see Table 5). The contradictory truth table rows^2^ resolved before minimizing the truth table algorithm.

**Table 5:** fsQCA intermediate solution for high life expectancy

Configuration	Raw coverage	Unique coverage	Consistency
EDUCATION AND GOVERNANCE AND HEALTH SYSTEM AND WEALTH	0.789	0.789	0.946
solution coverage: 0.789 solution consistency: 0.946			

The use of upper case for AND or OR means that they are being used as Boolean logical operators. In terms of fuzzy-set operations, the logical AND (*) - set intersection - refers to the minimum of each case’s membership in the combined sets [21]. For example, if a country’s membership in the set of high income countries is 0.6 and its membership in the set of countries that have good governance is 0.2, its membership in the set of countries that are rich and have good governance is the lowest of these two scores, i.e. 0.2.

The logical OR (+) - set union - refers to the maximum of each case’s membership in the combined sets [21]. For instance, if a country has a membership score of 0.35 in the set of high income countries and a membership score of 0.72 in the set of countries with good governance, it has a score of 0.72 in the set of countries that are rich or have good governance.

The fsQCA intermediate solution^3^, which tends to be the preferred solution due to its high interpretability [20], suggests that one configuration is consistent with usually being sufficient for high level life expectancy (see Table 5). The term ‘usually’ being sufficient is used since the level of consistency was not perfect (i.e. < 1) [31]. Table 5 indicates that the combination of high level of education AND high quality of governance AND affluent health system AND high wealth is the most empirically important path to high life expectancy. This configuration was found to be about 94% sufficient for providing high life expectancy and covered 79% of the membership scores in the outcome. A consistency score of 0.946 and a coverage score of 0.789 highlighted the high degree of theoretical significance and empirical importance of the solution terms.

Table 6 displays the membership scores of each case in the outcome of high life expectancy and the configuration resulting from the intermediate solution.

**Table 6:** Membership scores in the configuration linked to high life expectancy

Cases included in the configuration		E * G * H * W	High life expectancy
1	Japan	0.98	1
2	Spain	0.94	0.99
3	South Korea	0.94	0.99
4	Canada	0.97	0.99
5	France	0.98	0.99
6	Netherland	0.99	0.99
7	Sweden	0.99	0.99
8	Iceland	0.99	0.99
9	Austria	0.99	0.99
10	Switzerland	1	0.99
11	United kingdom	0.96	0.98
12	Finland	0.99	0.98
13	Australia	0.99	0.98
14	Ireland	0.99	0.98
15	Belgium	0.99	0.98
16	Norway	1	0.98
17	Germany	0.99	0.97
18	Denmark	1	0.97
19	United States	0.98	0.93
20	Czech Republic	0.95	0.9
	Raw coverage	0.789	
	Consistency	0.946	

*E* education, *G* governance, *H* health system, *I* income inequality, *W* wealth

Upper case: high level (> 0.5); Lower case: low level (< 0.5); *: AND

### What combination of structural conditions is usually sufficient for low life expectancy?

To answer the question above, an analysis of the low level life expectancy was conducted. Table 5 shows the truth table for all the configurations of socioeconomic conditions linked to the outcome of low life expectancy that included at least two empirical cases. Since the level of acceptable consistency was set at 0.9, eight configurations (in *italic*text) consistently explained the outcome.

The fuzzy-set truth table analysis summarized the eight relevant configurations and four causal recipes were identified. The intermediate solution in Table 7 gives four configurations that were usually sufficient to cause low life expectancy.

**Table 7:** fsQCA intermediate solution for low life expectancy

Configuration	Raw coverage	Unique coverage	Consistency
(governance AND income inequality) OR	0.401	0.045	0.957
(governance AND health system) OR	0.676	0.028	0.95
(education AND governance AND wealth) OR	0.679	0.019	0.971
(education AND health system AND INCOME INEQUALITY)	0.539	0..073	0.953
solution coverage: 0.863 solution consistency: 0.938			

Upper case: high level (> 0.5); Lower case: low level (< 0.5)

The overall consistency of this combination was found to be 93%. The recipe covered about 86% of the outcome.

As seen, the intermediate solutions resulting from the analysis of the five conditions in both directions (high/low) revealed combinations of conditions to be consistent with being usually sufficient to cause high/low life expectancy. These results implied that the potential causes of high or low life expectancy were configurational. This finding was consistent with the CSDH theoretical arguments.

The complex and parsimonious solutions are presented in Additional file 4.

## Sensitivity analysis

Since fsQCA is sensitive to researcher-specified thresholds and benchmarks related to 1) the calibration of raw data, 2) the minimum frequency of cases in each configuration, and 3) the consistency cutoff [12, 28], several robustness tests were conducted in this study. The crossover point varied ±10% for all conditions in both analyses [6, 30]. The lowest acceptable consistency for solutions was set at second-best choice that was 0.84. Finally, the minimum frequency threshold was set at one. The results reported above are robust to the use of these alternative specifications (see Additional file 5).

## Discussion

In this cross-national comparative analysis, countries were examined configurationally and different combinations of conditions linked to high/low life expectancy were identified. The first question in this research was “What combination of structural conditions is usually sufficient for high life expectancy?” The intermediate solutions resulted from fsQCA indicated that the combinations of high-quality governance, education, affluent health system, and high income were consistently sufficient to cause high life expectancy. It is interesting to note that this solution included a set of countries which all had high levels (≥ 0.9) of outcome of high life expectancy. It, hence, had high empirical relevance for the explanation of the outcome [20]. In response to the second question, we found that four configurations were consistently sufficient for low life expectancy. These results may provide an empirical support for the CSDH argument, which highlights the complexity of the interplay of contextual factors. The following sections will discuss the findings of the fsQCA in more details.

One of the most notable patterns that resulted from the fsQCA was related to the WHO European Region. This region contains 53 countries^4^ of which 46 were included in this study. The region faces issues of industrial and post-industrial society, as well as emerging democracies of central and Eastern Europe and the former Union of Soviet Socialist Republics (USSR) ([5, 16], Wilkinson, 2002). A close look at Table 4 shows that all European Union (EU) members, except Bulgaria, Romania, Latvia, and Lithuania, made up the configurations that were consistently sufficient for the outcome of high life expectancy (row 1 and 2). All of these countries, except Croatia, enjoyed high levels of education, governance, health system, wealth and low level of income inequality. Croatia, however, had a relatively high level of income inequality and was placed in row 2.

The former communist countries comprised another interesting group in this region. According to the fsQCA, the members of the former USSR in our sample, except Baltic states and Georgia (i.e. Armenia, Azerbaijan, Kazakhstan, Kyrgyz Republic, Moldova, Russian Federation, and Ukraine), along with other countries, made up the first configuration of intermediate solution for low life expectancy (see Table 8) This configuration indicated that weak governance in countries with a low level of income inequality was consistently sufficient for low life expectancy. Surprisingly, in this solution term, income inequality appeared in the unexpected theoretical direction. Nations that were officially ruled by a socialist ideology and were theoretically expected to have improved public health [3] had the lowest life expectancies in Europe [38]. Although the role of weak governance in the occurrence of low life expectancy was highlighted in this solution term, it must be noted that political conditions in countries are likely to rely on many structural and intermediary factors that might affect populations’ health. Nevertheless, such factors, e.g. weakness of the soviet health policy in dealing with social changes and crisis, environmental damage and occupational exposures (e.g. air pollution*),* and poor health behaviors including heavy alcohol drinking, smoking, physical inactivity, and unhealthy diet, were not incorporated in this study [3, 24]. Further in-depth research is thus needed to explain the mechanisms by which political conditions in such contexts lead to low life expectancy.

**Table 8:** Membership scores in the configuration linked to low life expectancy

Cases included in the configuration	g * i	g * h	e * g * w	e * h * I	Low life expectancy
Angola		0.96		0.96	1
Burundi	0.86	0.97	0.98		1
Cameroon		0.96	0.95		1
Chad		0.98	0.97		1
Côte d’Ivoire		0.98	0.95	0.88	1
Gambia				0.88	1
Ghana				0.84	1
Guinea		0.98	0.98	0.83	1
Liberia	0.67	0.98	0.96		1
Mozambique				0.97	1
Nigeria		0.97		0.91	1
Sierra Leone		0.96	0.96	0.81	1
Tanzania	0.58				1
Uganda			0.91		1
Zambia				0.92	1
Zimbabwe		0.93	0.94	0.93	1
Ethiopia	0.92	0.96	0.98		0.99
Kenya		0.95			0.99
Mauritania		0.97	0.94		0.99
Swaziland				0.86	0.99
Lao PDR	0.84	0.96	0.91		0.98
Madagascar			0.9	0.89	0.98
Namibia					0.98
Pakistan	0.99	0.96	0.92		0.98
Rwanda				0.95	0.98
Yemen	0.81		0.93		0.98
India	0.58				0.95
Timor-Leste	0.82		0.97		0.95
Cambodia		0.97	0.96		0.94
Indonesia	0.82				0.93
Nepal			0.97		0.92
Tajikistan	0.95				0.91
Belize				0.92	0.89
Kazakhstan	0.89				0.89
Russian Federation	0.94				0.88
Bolivia		0.92			0.87
Egypt	0.95				0.86
Kyrgyz Republic	0.82				0.85
Ukraine	0.91				0.84
Bangladesh		0.97	0.96		0.8
Guatemala		0.92		0.88	0.8
Moldova	0.75				0.78
Azerbaijan	0.97				0.74
El Salvador				0.84	0.67
Dominican Republic				0.83	0.63
Paraguay				0.91	0.62
Honduras		0.92		0.88	0.56
Armenia	0.58				0.54
Nicaragua			0.9	0.9	0.54
Sri Lanka	0.71				0.53
Raw coverage	0.401	0.676	0.679	0.539	
Consistency	0.957	0.95	0.971	0.953	

Baltic countries (i.e. Estonia, Lithuania, and Latvia) indicated the configuration of conditions linked to high life expectancy (see row 1 of Additional file 6). While the outcome was relatively high in Estonia, it was not as high as expected in Lithuania and Latvia and these two countries were more out of than in the set of countries with high life expectancy. They were both contradictory cases. The Baltic region witnessed dramatic socioeconomic and political changes following its independence during World War I and II. The USSR later reoccupied the region and the region switched to market economy in the early 1990s. It entered the EU in 2004 and found the chance to continue more favorable trends in life expectancy before Russia [35]. These major historical changes exerted strong positive/negative effects on the process of health transition in the region. Further in-depth analysis is, hence, required to explain the differences between the level of life expectancy in these three states where the outcome was not explained by the configuration detected here.

In contrast to the former USSR members, the former Yugoslavian Countries in our sample (i.e. Croatia, Bosnia and Herzegovina, Montenegro, Serbia, and Slovenia) and Albania had relatively high life expectancy. As can be seen in row 1 and 2 of Table 4, the combination of conditions that is consistent with being usually sufficient caused high life expectancy in Croatia, Montenegro, and Slovenia. In comparison, this study could not explain the relatively high level of life expectancy in Bosnia and Herzegovina, Serbia, and Albania by the condition used.

Overall, the relatively low life expectancy in Latvia, Lithuania (also Romania and Bulgaria), where the configuration was linked to high life expectancy, is a matter of question. It is also interesting how countries such as Bosnia and Herzegovina and Albania achieved a relatively high life expectancy, while they made up a configuration linked to the low level of outcome. These contradictory configurations indicated the presence of other causal pathways to the outcome of life expectancy. More detailed research would, thus, be required to identify the potentially causal conditions that could be added to the truth table analysis [20, 26]. The final report of the CSDH argued that health equity and well-being is influenced by a set of combinatory factors, including biological and psychosocial factors, the social environment, material circumstances, and behaviors. These factors are, in turn, affected by social position which is itself under the influence of gender, ethnicity, race, education, occupation, and income. All of such effects would definitely depend on the governing sociopolitical and sociocultural context [16, 29].

Another interesting place to explore is the American Region of the WHO. This region contains 35 countries^4^ of which 26 were included in our sample. As shown in row 1 and 2 of Table 4, Barbados, Canada, Uruguay, and the United States displayed configurations linked to the outcome of high life expectancy. However, a closer look at the data (see Additional file 1) indicated that some countries in this region had higher level of outcome but were not included in our identified configurations linked to high life expectancy. For instance, life expectancy is higher in Chile and Costa Rica than in the United States. However, the United States obviously outperformed both of them in wealth, education, quality of governance, and affluent health system. It would be interesting to study what other causally relevant conditions might contribute to the high level of life expectancy in Chile and Costa Rica. For instance, according to previous studies, in case of Costa Rica, one case-specific explanation might rely on lower socioeconomic gradients in the main causes of death (e.g. lung cancer and heart disease) compared to the United states ([25], Rosero-Bixby and Dow, 2016). Moreover, much steeper socioeconomic disparities were seen in the United States, compared to Costa Rica, for behavioral risk factors such as smoking, obesity, health insurance coverage, and uncontrolled dysglycemia and hypertension (Rosero-Bixby and Dow, 2016).

There were also some contradictory configurations in this region. The relatively high level of life expectancy in Panama, Mexico, Ecuador, Jamaica, and Peru and the relatively low level of life expectancy in Brazil were not explained by configurations detected in this study. Therefore, more in-depth research is warranted to explain their level of outcome.

The Western Pacific Region of WHO includes 27 countries^4^ of which 10 were recruited in our sample. Australia, Japan, and South Korea were cases included in the configuration that were consistently sufficient for high life expectancy. It should be noted that New Zealand and Singapore were cases of missing data. Two cases, i.e. China and Vietnam, had contradictory outcomes and their relatively high level of life expectancy could not be explained by this study. According to the existing literature, the great concern about socioeconomic determinants of health in both of these countries has led to the emergence of policy priorities for governance, especially regarding poverty reduction and access to health care for the poor [39]. Thus, a sole case study with more details on factors such as health gap reduction policies (e.g. targeted subsidies, investments in infrastructure, training at the grassroots level, disease prevention and health promotion in maternal and child health, and infectious diseases) may help explain their level of life expectancy [36, 39].

Malaysia is another country that may require a case study to explain its relatively low level of life expectancy. Malaysia made up the configuration of relatively high-level education, governance, and economic development combined with relatively poor health system and high income inequality. This configuration was not consistent for being sufficient to either high or low life expectancy (see row 5 of Table 4).

The fsQCA indicated that among the six WHO regions, none of the Eastern Mediterranean, South-East Asian, and African countries in the sample were included in configurations that were consistently sufficient for high life expectancy. However, our study could not help to explain the level of life expectancy in Iran and Tunisia. They indicated configurations linked to low life expectancy (see row 7 and 8 of Additional file 6), while there were more in than out in the set of countries with high life expectancy. Due to such contradictions, we excluded these countries from the sample [26].

## Conclusion

The primary goal of this configurational comparative study was to assess the connection between combinations of socioeconomic conditions and life expectancy at birth and to compare the results with the CSDH conceptual framework. The results of the fsQCA indicated that high or low life expectancy could be affected by combination of socioeconomic and political conditions. These results supported the CSDH’s argument that policies and actions on health, as a complex phenomenon, should be intersectoral in nature. Critics of QCA would argue that fsQCA is made to find configurational effects even where none may exist in reality [13]. Yet, Ragin stated that QCA privileges complexity, while conventional quantitative methods privilege simplicity. When substantive knowledge tells us that causation is complex or combinatorial, it is very unlikely to identify it using conventional quantitative methods. Indeed, what exists in reality is best described by substantive knowledge and is not the property of an analytical method [23].

According to the assessment of the combinations of conditions leading to high or low life expectancy, the configurations linked to high life expectancy were not the opposite of those associated with low life expectancy (see Table 5 and Table 7). This is in accordance with the concept of asymmetric causality [20].

Overall, this study evaluated the configurational effects of structural conditions, instead of estimating the “net effect” of each factor on life expectancy. As Ragin noted, “policy discourse often focuses on categories and kinds of people (or cases), not on variables and their net effects across heterogeneous populations” [20]. While it is useful to assess the impact of socioeconomic factors on population health, it could be more helpful for policymakers to know under what conditions such factors play a role. As discussed, a context-specific assessment is needed to answer such questions and this is possible when cases are studied as configurations (as done in this study) [20].

Finally, based on our findings, further in-depth research and detailed examinations are required in particular countries to explain their levels of life expectancy.

## Limitations

This research had some limitations regarding problems of causal ambiguity, operationalization, and generalizability.

The most notable limitation was the problem of causal ambiguity. Even though we used the fsQCA as an appropriate methodological tool for analyzing causal complexity, we could not prove causality. fsQCA identifies configurations that are consistent with usually being sufficient to cause outcomes. It does not eliminate the chance of spurious conjunction of configurations and outcomes [30]. Moreover, while health is mainly affected by the socioeconomic position of individuals [15, 18, 29], it, in some cases, causes social and economic consequences, i.e. it actually determines individuals’ socioeconomic position [29].

Since such conditions may lead to health inequality, there is still a chance of reverse causation (see Fig. 1).

Table 4 shows that a combination of all five conditions was consistently sufficient for high life expectancy (row 2). This could be the result of a mathematical quirk of the fsQCA, rather than an indicator of real causation. As mentioned earlier, according to the way that logical AND accomplished, creating a combination of unions of all five conditions meant that each case entered the analysis with its lowest possible configurational set score. The analysis of sufficiency in fsQCA then tested whether the cases’ scores for given configuration were equal to or lower than their scores for the outcome condition. Thus, larger number of conditions included in the solution would increase the risk of the sufficiency test yielding a spurious result. It is worth noting again that substantive knowledge is of the greatest importance. If the five-condition recipe made good sense and could be connected to cases, these facts undercut the argument that the recipe is spurious. However, as recommended by Ragin, we computed the degree of membership in each case’s second lowest membership score (of the five conditions). By comparing consistency and coverage scores, we found that having four out of five conditions was good enough to explain the outcome of high life expectancy.

The next challenge in this analysis was related to the operationalization and measurement. We were not certain if it was theoretically justifiable to give equal weight to all the sub-components of the scales used to measure the components (e.g. quality of governance and health system). Ideally, each sub-component had to be assigned a weight indicating the level of importance it had in affecting life expectancy.

Another limitation of the data used in this research was the selection biases that decreased the generalizability of the results. As mentioned earlier, cases with missing data were removed from the study sample. For instance, all members of the Gulf Cooperation Council (i.e. Bahrain, Kuwait, Oman, Qatar, Saudi Arabia, and the United Arab Emirates) were excluded. Some contradictory cases were also removed from the sample. Excluding these cases, which might have been systematically different from those included in the sample, decreased the generalizability of the results [30].

## Notes

1. In our preliminary analysis, based on the first element of the CSDH framework, which refers to “socioeconomic context and structural determinants” and available national data, we focused on seven conditions including governance, economic development, education, labor market efficiency, social protection, and two intermediary factors including health system and social capital. Considering the large amount of missing data in the social protection indicators extracted from the WDI, related data were not included in the analysis as their inclusion would have reduced the sample size. Labor market efficiency, extracted from the GCI, and social capital, derived from the Legatum Prosperity Index™, were also eliminated from the analysis because they were found irrelevant to the theoretically expected association between these factors and the outcome. Furthermore, the model would have become more complex by adding these two conditions to the analysis.
2. Contradictory rows showed configurations of conditions which contained cases with different outcomes [20, 26]. In this study, there were some contradictory cases that indicated the same combination of conditions, but were different in the outcome detected (see Additional file 6). To resolve contradictions, firstly, the thresholds for calibration of the outcome were adjusted to an extent that they remained consistent with substantive knowledge of the cases. Secondly, the case selection was modified to exclude the contradictory cases from the sample [26]. However, we reported them in the discussion because future more detailed investigations of these particular cases would probably yield relevant causal conditions that could be added to the analysis [20, 26]
3. Assumptions for generating the intermediate solution for high life expectancy (made based on the existing theoretical knowledge) were the presence of high level of education, governance, health system, and wealth and the absence of high level of income inequality. The opposite of these assumptions were applied in developing the intermediate solution for low life expectancy.
4. http://www.who.int/about/regions/en/
  ******.

Please find the link below for the data that support the findings of this study.

## Additional files

Additional file 1: Raw Data. (DOCX 66 kb)

Additional file 2: Interval-scales and indicators. (DOCX 37 kb)

Additional file 3: Fuzzy-set scores (Original calibration). (DOCX 61 kb)

Additional file 4: fsQCA Complex and Parsimonious solutions. (DOCX 40 kb)

Additional file 5: Sensitivity analysis. (DOCX 71 kb)

Additional file 6: Distribution of cases across combinations of causal conditions (Contradictory cases are shown in bold text). (DOCX 22 kb)

## Abbreviations

CSDH: Commission on Social Determinants of Health
GCI: Global Competitiveness Index
SDH: Social Determinants of Health
USSR: The Union of Soviet Socialist Republics
WGI: Worldwide Governance Indicator
WHO: World Health Organization
