# Supplementary material for: A fuzzy set qualitative comparative analysis of 131 countries: which configuration of the structural conditions can explain health better?
Source: Int J Equity Health. 2018 Jan 22;17:10. doi: 10.1186/s12939-018-0724-1 (PMC5778742; doi:10.1186/s12939-018-0724-1)
Supplement: Supplementary file 1 — Raw Data. (DOCX 66 kb) [file 12939_2018_724_MOESM1_ESM.docx]

Additional file 2 Raw Data

| **Row** | **Attribute** | **Code** | **WHO Region** | **Life expectancy** | **Education** | **Governance** | **Health System** | **Income inequality** | **Wealth** |
| --- | --- | --- | --- | --- | --- | --- | --- | --- | --- |
| 1 | Albania | ALB | European Region | 77.8 | 3.85 | -0.23 | -0.24 | 17.52 | 9569 |
| 2 | Angola | AGO | African Region | 52.4 | 2.01 | -1.07 | -0.75 | 43.06 | 5669 |
| 3 | Argentina | ARG | Region of the Americas | 76.3 | 4.48 | -0.3 | 0.16 | 31.52 | 18233 |
| 4 | Armenia | ARM | European Region | 74.8 | 3.82 | -0.26 | 0.17 | 12.74 | 7062 |
| 5 | Australia | AUS | Western Pacific Region | 82.8 | 5.56 | 1.6 | 1.38 | 16.82 | 41576 |
| 6 | Austria | AUT | European Region | 81.5 | 5.43 | 1.57 | 1.88 | 14.44 | 43459 |
| 7 | Azerbaijan | AZE | European Region | 72.7 | 3.83 | -0.78 | 0.75 | 5.36 | 14488 |
| 8 | Bangladesh | BGD | South-East Asian Region | 71.8 | 2.71 | -0.9 | -0.86 | 21.36 | 2488 |
| 9 | Barbados | BRB | Region of the Americas | 75.5 | 5.04 | 1.16 | 0.57 | 16.1 | 15513 |
| 10 | Belgium | BEL | European Region | 81.1 | 5.73 | 1.31 | 1.75 | 12.52 | 41034 |
| 11 | Belize | BLZ | Region of the Americas | 70.1 | 3.2 | -0.12 | -0.58 | 41.43 | 7934 |
| 12 | Benin | BEN | African Region | 60 | 2.99 | -0.3 | -0.85 | 23.88 | 1853 |
| 13 | Bhutan | BTN | South-East Asian Region | 69.8 | 3.49 | 0.17 | -0.69 | 22.6 | 6233 |
| 14 | Bolivia | BOL | Region of the Americas | 70.7 | 3.61 | -0.6 | -0.72 | 44.2 | 5520 |
| 15 | Bosnia and Herzegovina | BIH | European Region | 77.4 | 3.71 | -0.34 | -0.01 | 21.8 | 9723 |
| 16 | Botswana | BWA | African Region | 65.7 | 3.62 | 0.68 | -0.46 | 55.5 | 13791 |
| 17 | Brazil | BRA | Region of the Americas | 75 | 4.23 | -0.03 | -0.04 | 39.28 | 14161 |
| 18 | Bulgaria | BGR | European Region | 74.5 | 4.2 | 0.18 | 0.62 | 17.6 | 15279 |
| 19 | Burkina Faso | BFA | African Region | 59.9 | 2.51 | -0.37 | -0.84 | 24.48 | 1413 |
| 20 | Burundi | BDI | African Region | 59.6 | 2.17 | -1.12 | -0.78 | 17.4 | 762 |
| 21 | Cambodia | KHM | Western Pacific Region | 68.7 | 2.87 | -0.8 | -0.83 | 20.74 | 2608 |
| 22 | Cameroon | CMR | African Region | 57.3 | 3.04 | -0.9 | -0.73 | 21.18 | 2732 |
| 23 | Canada | CAN | Region of the Americas | 82.2 | 5.53 | 1.61 | 1.1 | 16.52 | 41562 |
| 24 | Cape Verde | CPV | African Region | 73.3 | 3.68 | 0.48 | -0.6 | 28 | 5690 |
| 25 | Chad | TCD | African Region | 53.1 | 2.16 | -1.36 | -0.89 | 22.9 | 1877 |
| 26 | Chile | CHL | Region of the Americas | 80.5 | 4.66 | 1.17 | -0.38 | 34.86 | 19911 |
| 27 | China | CHN | Western Pacific Region | 76.1 | 4.14 | -0.52 | -0.25 | 29.5 | 9577 |
| 28 | Colombia | COL | Region of the Americas | 74.8 | 4.12 | -0.36 | -0.53 | 44.18 | 11156 |
| 29 | Costa Rica | CRI | Region of the Americas | 79.6 | 4.61 | 0.57 | -0.5 | 34.78 | 13101 |
| 30 | Côte d'Ivoire | CIV | African Region | 53.3 | 3.09 | -1.08 | -0.86 | 28.96 | 2771 |
| 31 | Croatia | HRV | European Region | 78 | 4.41 | 0.39 | 0.55 | 23.64 | 20554 |
| 32 | Cyprus | CYP | European Region | 80.5 | 4.8 | 1.05 | 0.38 | 13.64 | 32986 |
| 33 | Czech Republic | CZE | European Region | 78.8 | 4.97 | 0.89 | 1.11 | 10.96 | 28408 |
| 34 | Denmark | DNK | European Region | 80.6 | 5.8 | 1.82 | 1.75 | 12.04 | 44929 |
| 35 | Dominican Republic | DOM | Region of the Americas | 73.9 | 3.53 | -0.33 | -0.54 | 32.72 | 11028 |
| 36 | Ecuador | ECU | Region of the Americas | 76.2 | 3.55 | -0.75 | -0.41 | 32.8 | 9705 |
| 37 | Egypt | EGY | Eastern Mediterranean Region | 70.9 | 3.46 | -0.67 | -0.27 | 14.54 | 9400 |
| 38 | El Salvador | SLV | Region of the Americas | 73.5 | 3.51 | -0.14 | -0.6 | 32.58 | 7434 |
| 39 | Estonia | EST | European Region | 77.6 | 5.24 | 1.06 | 0.64 | 17.18 | 25187 |
| 40 | Ethiopia | ETH | African Region | 64.8 | 2.62 | -0.93 | -0.72 | 16.28 | 1094 |
| 41 | Finland | FIN | European Region | 81.1 | 6.11 | 1.85 | 1.67 | 11.38 | 40132 |
| 42 | France | FRA | European Region | 82.4 | 5.31 | 1.21 | 1.63 | 13.84 | 37249 |
| 43 | Gabon | GAB | African Region | 66 | 2.81 | -0.56 | -0.23 | 21.42 | 16070 |
| 44 | Gambia, The | GMB | African Region | 61.1 | 3.39 | -0.54 | -0.79 | 29.03 | 1568 |
| 45 | Georgia | GEO | European Region | 74.4 | 3.76 | -0.01 | 0.19 | 24.76 | 7013 |
| 46 | Germany | DEU | European Region | 81 | 5.48 | 1.48 | 1.92 | 14.92 | 41196 |
| 47 | Ghana | GHA | African Region | 62.4 | 3.35 | 0.06 | -0.78 | 27.74 | 3209 |
| 48 | Greece | GRC | European Region | 81 | 4.65 | 0.44 | 0.96 | 18.38 | 27831 |
| 49 | Guatemala | GTM | Region of the Americas | 71.9 | 3.39 | -0.6 | -0.7 | 39.82 | 6800 |
| 50 | Guinea | GIN | African Region | 59 | 2.35 | -1.23 | -0.89 | 27.44 | 1209 |
| 51 | Guyana | GUY | Region of the Americas | 66.2 | 3.77 | -0.4 | -0.56 | 25.12 | 5903 |
| 52 | Honduras | HND | Region of the Americas | 74.6 | 3.39 | -0.61 | -0.74 | 42.86 | 4038 |
| 53 | Hungary | HUN | European Region | 75.9 | 4.68 | 0.73 | 0.93 | 12.76 | 23080 |
| 54 | Iceland | ISL | European Region | 82.7 | 5.65 | 1.58 | 1.66 | 12.44 | 40981 |
| 55 | India | IND | South-East Asian Region | 68.3 | 3.97 | -0.26 | -0.71 | 15.48 | 4384 |
| 56 | Indonesia | IDN | South-East Asian Region | 69.1 | 4.14 | -0.45 | -0.77 | 17.04 | 8523 |
| 57 | Iran, Islamic Rep. | IRN | Eastern Mediterranean Region | 75.5 | 4.04 | -1.06 | -0.57 | 46.6 | 16303 |
| 58 | Ireland | IRL | European Region | 81.4 | 5.31 | 1.5 | 1.61 | 15.2 | 48536 |
| 59 | Israel | ISR | European Region | 82.5 | 5.05 | 0.6 | 0.76 | 23.14 | 29612 |
| 60 | Italy | ITA | European Region | 82.7 | 4.63 | 0.53 | 0.8 | 18.78 | 36210 |
| 61 | Jamaica | JAM | Region of the Americas | 76.2 | 3.96 | 0 | -0.58 | 27.7 | 8247 |
| 62 | Japan | JPN | Western Pacific Region | 83.7 | 5.27 | 1.25 | 2.34 | 13.5 | 36298 |
| 63 | Jordan | JOR | Eastern Mediterranean Region | 74.1 | 4.46 | -0.04 | -0.24 | 20.5 | 9131 |
| 64 | Kazakhstan | KAZ | European Region | 70.2 | 4.29 | -0.54 | 0.91 | 14.82 | 20377 |
| 65 | Kenya | KEN | African Region | 63.4 | 3.64 | -0.68 | -0.72 | 34.56 | 2501 |
| 66 | Korea, Rep. | KOR | Western Pacific Region | 82.3 | 5.43 | 0.75 | 1.1 | 18.4 | 30088 |
| 67 | Kyrgyz Republic | KGZ | European Region | 71.1 | 3.73 | -0.87 | 0.24 | 18.06 | 2830 |
| 68 | Lao PDR | LAO | Western Pacific Region | 65.7 | 3.28 | -0.92 | -0.74 | 17.74 | 4074 |
| 69 | Latvia | LVA | European Region | 74.6 | 4.85 | 0.67 | 0.69 | 20.54 | 20440 |
| 70 | Lebanon | LBN | Eastern Mediterranean Region | 74.9 | 4.59 | -0.69 | 0.06 | 30 | 14134 |
| 71 | Liberia | LBR | African Region | 61.4 | 2.98 | -0.88 | -0.85 | 19.74 | 714 |
| 72 | Lithuania | LTU | European Region | 73.6 | 5.06 | 0.75 | 0.96 | 18.86 | 22880 |
| 73 | Luxembourg | LUX | European Region | 82 | 4.65 | 1.7 | 2.13 | 13.56 | 92322 |
| 74 | Madagascar | MDG | African Region | 65.5 | 2.69 | -0.56 | -0.89 | 29.6 | 1405 |
| 75 | Malawi | MWI | African Region | 58.3 | 2.76 | -0.36 | -0.78 | 22.96 | 992 |
| 76 | Malaysia | MYS | Western Pacific Region | 75 | 4.74 | 0.36 | -0.4 | 28.7 | 21593 |
| 77 | Mali | MLI | African Region | 58.2 | 2.66 | -0.5 | -0.89 | 20.75 | 1827 |
| 78 | Malta | MLT | European Region | 81.7 | 4.72 | 1.17 | 0.89 | 13.67 | 29038 |
| 79 | Mauritania | MRT | African Region | 63.1 | 2.23 | -0.77 | -0.86 | 21.84 | 3410 |
| 80 | Mauritius | MUS | African Region | 74.6 | 4.18 | 0.8 | -0.17 | 18.2 | 15867 |
| 81 | Mexico | MEX | Region of the Americas | 76.7 | 3.96 | -0.17 | -0.28 | 34.4 | 15941 |
| 82 | Moldova | MDA | European Region | 72.1 | 3.98 | -0.39 | 0.53 | 17.64 | 4017 |
| 83 | Mongolia | MNG | Western Pacific Region | 68.8 | 3.95 | -0.16 | 0.36 | 15.42 | 8581 |
| 84 | Montenegro | MNE | European Region | 76.1 | 4.42 | 0 | 0.13 | 12.08 | 13902 |
| 85 | Morocco | MAR | Eastern Mediterranean Region | 74.3 | 3.53 | -0.33 | -0.7 | 22.54 | 6415 |
| 86 | Mozambique | MOZ | African Region | 57.6 | 2.42 | -0.38 | -0.84 | 35.54 | 928 |
| 87 | Namibia | NAM | African Region | 65.8 | 3.17 | 0.32 | -0.32 | 68.3 | 8574 |
| 88 | Nepal | NPL | South-East Asian Region | 69.2 | 2.81 | -0.85 | -0.25 | 26.92 | 1980 |
| 89 | Netherlands | NLD | European Region | 81.9 | 5.71 | 1.67 | 1.73 | 12.7 | 45589 |
| 90 | Nicaragua | NIC | Region of the Americas | 74.8 | 3.17 | -0.56 | -0.68 | 30.06 | 4247 |
| 91 | Nigeria | NGA | African Region | 54.5 | 3.02 | -1.13 | -0.77 | 30.26 | 5024 |
| 92 | Norway | NOR | European Region | 81.8 | 5.62 | 1.72 | 2.31 | 11.48 | 63277 |
| 93 | Pakistan | PAK | Eastern Mediterranean Region | 66.4 | 2.84 | -1.06 | -0.75 | 11.04 | 4341 |
| 94 | Panama | PAN | Region of the Americas | 77.8 | 4 | 0.1 | -0.3 | 41.18 | 16213 |
| 95 | Paraguay | PRY | Region of the Americas | 74 | 3.05 | -0.67 | -0.55 | 32.98 | 7264 |
| 96 | Peru | PER | Region of the Americas | 75.5 | 3.89 | -0.28 | -0.56 | 33.54 | 9912 |
| 97 | Philippines | PHL | Western Pacific Region | 68.5 | 4.16 | -0.42 | -0.47 | 28 | 5687 |
| 98 | Poland | POL | European Region | 77.5 | 4.86 | 0.72 | 0.48 | 17.44 | 21583 |
| 99 | Portugal | PRT | European Region | 81.1 | 4.87 | 1 | 0.6 | 20.84 | 26683 |
| 100 | Romania | ROM | European Region | 75 | 4.37 | 0.12 | 0.47 | 18.2 | 18017 |
| 101 | Russian Federation | RUS | European Region | 70.5 | 4.56 | -0.72 | 1.33 | 15.46 | 23221 |
| 102 | Rwanda | RWA | African Region | 66.1 | 3.04 | -0.34 | -0.71 | 34.2 | 1363 |
| 103 | Senegal | SEN | African Region | 66.7 | 3.25 | -0.25 | -0.91 | 23.4 | 2172 |
| 104 | Serbia | SRB | European Region | 75.6 | 3.99 | -0.18 | 0.31 | 11.96 | 12573 |
| 105 | Sierra Leone | SLE | African Region | 50.1 | 2.4 | -0.74 | -0.89 | 26.88 | 1283 |
| 106 | Slovak Republic | SVK | European Region | 76.7 | 4.49 | 0.75 | 0.81 | 11.08 | 24948 |
| 107 | Slovenia | SVN | European Region | 80.8 | 5.21 | 0.93 | 0.71 | 10.44 | 28706 |
| 108 | South Africa | ZAF | African Region | 62.9 | 4.06 | 0.28 | -0.64 | 49.1 | 12049 |
| 109 | Spain | ESP | European Region | 82.8 | 4.93 | 0.86 | 0.82 | 20.18 | 32501 |
| 110 | Sri Lanka | LKA | South-East Asian Region | 74.9 | 4.06 | -0.35 | -0.37 | 19.14 | 8760 |
| 111 | Suriname | SUR | Region of the Americas | 71.6 | 3.53 | -0.12 | -0.1 | 35.44 | 14042 |
| 112 | Swaziland | SWZ | African Region | 58.9 | 2.99 | -0.6 | -0.45 | 37.86 | 7417 |
| 113 | Sweden | SWE | European Region | 82.4 | 5.79 | 1.76 | 1.4 | 12 | 43242 |
| 114 | Switzerland | CHE | European Region | 83.4 | 5.78 | 1.74 | 2.5 | 13.68 | 55458 |
| 115 | Tajikistan | TJK | European Region | 69.7 | 3.5 | -1.1 | 0.26 | 15.12 | 2151 |
| 116 | Tanzania | TZA | African Region | 61.8 | 2.54 | -0.4 | -0.8 | 20.54 | 2122 |
| 117 | Thailand | THA | South-East Asian Region | 74.9 | 4.37 | -0.27 | -0.53 | 34 | 13442 |
| 118 | Timor-Leste | TMP | South-East Asian Region | 68.3 | 2.63 | -0.8 | -0.12 | 18.08 | 1756 |
| 119 | Trinidad and Tobago | TTO | Region of the Americas | 71.2 | 4.08 | 0.12 | -0.15 | 21.9 | 30722 |
| 120 | Tunisia | TUN | Eastern Mediterranean Region | 75.3 | 4.57 | -0.17 | -0.38 | 20.83 | 10084 |
| 121 | Turkey | TUR | European Region | 75.8 | 4.16 | -0.06 | -0.26 | 24.62 | 19220 |
| 122 | Uganda | UGA | African Region | 62.3 | 2.78 | -0.59 | 0.98 | 27.84 | 1522 |
| 123 | Ukraine | UKR | European Region | 71.3 | 4.58 | -0.58 | 0.26 | 10.46 | 8024 |
| 124 | United Kingdom | GBR | European Region | 81.2 | 5.43 | 1.42 | 0.93 | 18.36 | 37199 |
| 125 | United States | USA | Region of the Americas | 79.3 | 5.71 | 1.25 | 1.78 | 30.24 | 50512 |
| 126 | Uruguay | URY | Region of the Americas | 77 | 4.44 | 0.77 | 1.78 | 26.66 | 16763 |
| 127 | Venezuela | VEN | Region of the Americas | 74.1 | 4.03 | -1.24 | -0.41 | 35.72 | 16911 |
| 128 | Vietnam | VNM | Western Pacific Region | 76 | 3.56 | -0.51 | -0.8 | 15.52 | 4518 |
| 129 | Yemen | YEM | Eastern Mediterranean Region | 65.7 | 2.31 | -1.25 | -0.2 | 18.2 | 3943 |
| 130 | Zambia | ZMB | African Region | 61.8 | 3.13 | -0.33 | -0.59 | 33.88 | 3161 |
| 131 | Zimbabwe | ZWE | African Region | 60.7 | 3.13 | -1.46 | -0.63 | 35.28 | 1627 |
