# Supplementary material for: A fuzzy set qualitative comparative analysis of 131 countries: which configuration of the structural conditions can explain health better?
Source: Int J Equity Health. 2018 Jan 22;17:10. doi: 10.1186/s12939-018-0724-1 (PMC5778742; doi:10.1186/s12939-018-0724-1)
Supplement: Supplementary file 2 — Interval-scales and indicators. (DOCX 37 kb) [file 12939_2018_724_MOESM2_ESM.docx]

Additional file 1 Interval-scales and indicators

| Interval-scale variable | Data Source | Period | Indicators |
| --- | --- | --- | --- |
| Education | The 5th pillar of the Global Competitiveness Index (GCI),World Economic Forum | 2005-2015 | Quantity of education  Secondary education enrollment, gross %  Tertiary education enrollment, gross %  Quality of education  Quality of the education system  Quality of math and science education  Quality of management schools  Internet access in schools  On-the-job training  Availability of research and training services  Extent of staff training |
| Governance | The Worldwide Governance Indicator (WGI), World Bank | 2004-2014 | Voice & Accountability  Political Stability and Lack of Violence Government Effectiveness  Regulatory Quality  Rule of Law  Control of Corruption. |
| Health System | World Bank | 2004-2014 | Health workforce  Density of physicians (per 1000 population)  Density of nursing and midwifery personnel (per 1000 population)  Infrastructure  Hospitals Bed (per 1000 population)  Health expenditure per capita (current US$) |
| Income inequality | United Nations’ Human Development Index | 2010-2015 | Atkinson’s measure of income inequality |
| Wealth | World Bank | 2005-2015 | GDP per capita, PPP (constant 2011 international $) |
|  |  |  |  |
