# Supplementary material for: A fuzzy set qualitative comparative analysis of 131 countries: which configuration of the structural conditions can explain health better?
Source: Int J Equity Health. 2018 Jan 22;17:10. doi: 10.1186/s12939-018-0724-1 (PMC5778742; doi:10.1186/s12939-018-0724-1)
Supplement: Supplementary file 3 — Fuzzy-set scores (Original calibration). (DOCX 61 kb) [file 12939_2018_724_MOESM3_ESM.docx]

Additional file 3 Fuzzy-set scores (Original calibration)

| **Row** | **Countries** | **Code** | **WHO Region** | **High education** | **Good governance** | **Affluent health system** | **High income inequality** | **High wealth** | **High life exp.** | **Low life exp.** |
| --- | --- | --- | --- | --- | --- | --- | --- | --- | --- | --- |
| 1 | Albania | ALB | European Region | 0.33 | 0.47 | 0.32 | 0.14 | 0.41 | 0.84 | 0.16 |
| 2 | Angola | AGO | African Region | 0 | 0 | 0.04 | 1 | 0.13 | 0 | 1 |
| 3 | Argentina | ARG | Region of the Americas | 0.82 | 0.36 | 0.68 | 0.93 | 0.78 | 0.67 | 0.33 |
| 4 | Armenia | ARM | European Region | 0.32 | 0.42 | 0.68 | 0.02 | 0.2 | 0.46 | 0.54 |
| 5 | Australia | AUS | Western Pacific Region | 1 | 0.99 | 0.99 | 0.11 | 0.99 | 0.99 | 0.01 |
| 6 | Austria | AUT | European Region | 0.99 | 0.99 | 1 | 0.04 | 1 | 0.98 | 0.02 |
| 7 | Azerbaijan | AZE | European Region | 0.32 | 0.03 | 0.93 | 0 | 0.66 | 0.26 | 0.74 |
| 8 | Bangladesh | BGD | South-East Asian Region | 0.02 | 0.01 | 0.02 | 0.51 | 0.04 | 0.2 | 0.8 |
| 9 | Barbados | BRB | Region of the Americas | 0.98 | 0.98 | 0.88 | 0.08 | 0.7 | 0.55 | 0.45 |
| 10 | Belgium | BEL | European Region | 1 | 0.99 | 1 | 0.01 | 0.99 | 0.98 | 0.02 |
| 11 | Belize | BLZ | Region of the Americas | 0.07 | 0.56 | 0.08 | 0.99 | 0.26 | 0.11 | 0.89 |
| 12 | Benin | BEN | African Region | 0.04 | 0.36 | 0.02 | 0.66 | 0.03 | 0 | 1 |
| 13 | Bhutan | BTN | South-East Asian Region | 0.15 | 0.74 | 0.05 | 0.59 | 0.15 | 0.1 | 0.9 |
| 14 | Bolivia | BOL | Region of the Americas | 0.2 | 0.08 | 0.04 | 1 | 0.12 | 0.13 | 0.87 |
| 15 | Bosnia and Herzegovina | BIH | European Region | 0.25 | 0.31 | 0.55 | 0.54 | 0.42 | 0.8 | 0.2 |
| 16 | Botswana | BWA | African Region | 0.21 | 0.92 | 0.14 | 1 | 0.63 | 0.02 | 0.98 |
| 17 | Brazil | BRA | Region of the Americas | 0.63 | 0.62 | 0.53 | 0.99 | 0.65 | 0.48 | 0.52 |
| 18 | Bulgaria | BGR | European Region | 0.61 | 0.75 | 0.89 | 0.15 | 0.69 | 0.43 | 0.57 |
| 19 | Burkina Faso | BFA | African Region | 0.01 | 0.27 | 0.02 | 0.7 | 0.03 | 0 | 1 |
| 20 | Burundi | BDI | African Region | 0 | 0 | 0.03 | 0.14 | 0.02 | 0 | 1 |
| 21 | Cambodia | KHM | Western Pacific Region | 0.03 | 0.02 | 0.03 | 0.44 | 0.04 | 0.06 | 0.94 |
| 22 | Cameroon | CMR | African Region | 0.05 | 0.01 | 0.04 | 0.49 | 0.04 | 0 | 1 |
| 23 | Canada | CAN | Region of the Americas | 1 | 0.99 | 0.97 | 0.09 | 0.99 | 0.99 | 0.01 |
| 24 | Cape Verde | CPV | African Region | 0.24 | 0.87 | 0.07 | 0.85 | 0.13 | 0.31 | 0.69 |
| 25 | Chad | TCD | African Region | 0 | 0 | 0.02 | 0.6 | 0.03 | 0 | 1 |
| 26 | Chile | CHL | Region of the Americas | 0.9 | 0.98 | 0.19 | 0.97 | 0.83 | 0.96 | 0.04 |
| 27 | China | CHN | Western Pacific Region | 0.55 | 0.13 | 0.31 | 0.89 | 0.41 | 0.64 | 0.36 |
| 28 | Colombia | COL | Region of the Americas | 0.53 | 0.28 | 0.1 | 1 | 0.53 | 0.46 | 0.54 |
| 29 | Costa Rica | CRI | Region of the Americas | 0.88 | 0.9 | 0.12 | 0.97 | 0.61 | 0.94 | 0.06 |
| 30 | Côte d'Ivoire | CIV | African Region | 0.05 | 0 | 0.02 | 0.88 | 0.04 | 0 | 1 |
| 31 | Croatia | HRV | European Region | 0.78 | 0.84 | 0.87 | 0.65 | 0.84 | 0.85 | 0.15 |
| 32 | Cyprus | CYP | European Region | 0.94 | 0.97 | 0.8 | 0.03 | 0.98 | 0.96 | 0.04 |
| 33 | Czech Republic | CZE | European Region | 0.97 | 0.96 | 0.97 | 0.01 | 0.95 | 0.9 | 0.1 |
| 34 | Denmark | DNK | European Region | 1 | 1 | 1 | 0.01 | 1 | 0.97 | 0.03 |
| 35 | Dominican Republic | DOM | Region of the Americas | 0.17 | 0.32 | 0.1 | 0.95 | 0.52 | 0.37 | 0.63 |
| 36 | Ecuador | ECU | Region of the Americas | 0.18 | 0.03 | 0.17 | 0.95 | 0.42 | 0.65 | 0.35 |
| 37 | Egypt | EGY | Eastern Mediterranean Region | 0.14 | 0.05 | 0.29 | 0.04 | 0.39 | 0.14 | 0.86 |
| 38 | El Salvador | SLV | Region of the Americas | 0.16 | 0.55 | 0.07 | 0.95 | 0.23 | 0.33 | 0.67 |
| 39 | Estonia | EST | European Region | 0.99 | 0.97 | 0.9 | 0.12 | 0.92 | 0.82 | 0.18 |
| 40 | Ethiopia | ETH | African Region | 0.01 | 0.01 | 0.04 | 0.08 | 0.02 | 0.01 | 0.99 |
| 41 | Finland | FIN | European Region | 1 | 1 | 1 | 0.01 | 0.99 | 0.98 | 0.02 |
| 42 | France | FRA | European Region | 0.99 | 0.98 | 0.99 | 0.03 | 0.99 | 0.99 | 0.01 |
| 43 | Gabon | GAB | African Region | 0.03 | 0.1 | 0.33 | 0.51 | 0.72 | 0.02 | 0.98 |
| 44 | Gambia, The | GMB | African Region | 0.12 | 0.11 | 0.03 | 0.88 | 0.03 | 0 | 1 |
| 45 | Georgia | GEO | European Region | 0.28 | 0.64 | 0.7 | 0.71 | 0.2 | 0.42 | 0.58 |
| 46 | Germany | DEU | European Region | 1 | 0.99 | 1 | 0.05 | 0.99 | 0.97 | 0.03 |
| 47 | Ghana | GHA | African Region | 0.11 | 0.68 | 0.03 | 0.84 | 0.05 | 0 | 1 |
| 48 | Greece | GRC | European Region | 0.9 | 0.86 | 0.96 | 0.2 | 0.95 | 0.97 | 0.03 |
| 49 | Guatemala | GTM | Region of the Americas | 0.12 | 0.08 | 0.05 | 0.99 | 0.19 | 0.2 | 0.8 |
| 50 | Guinea | GIN | African Region | 0.01 | 0 | 0.02 | 0.83 | 0.02 | 0 | 1 |
| 51 | Guyana | GUY | Region of the Americas | 0.29 | 0.23 | 0.09 | 0.73 | 0.14 | 0.02 | 0.98 |
| 52 | Honduras | HND | Region of the Americas | 0.12 | 0.08 | 0.04 | 1 | 0.07 | 0.44 | 0.56 |
| 53 | Hungary | HUN | European Region | 0.91 | 0.93 | 0.96 | 0.02 | 0.89 | 0.61 | 0.39 |
| 54 | Iceland | ISL | European Region | 1 | 0.99 | 1 | 0.01 | 0.99 | 0.99 | 0.01 |
| 55 | India | IND | South-East Asian Region | 0.42 | 0.42 | 0.05 | 0.06 | 0.08 | 0.05 | 0.95 |
| 56 | Indonesia | IDN | South-East Asian Region | 0.55 | 0.18 | 0.03 | 0.12 | 0.31 | 0.07 | 0.93 |
| 57 | Iran, Islamic Rep. | IRN | Eastern Mediterranean Region | 0.46 | 0 | 0.09 | 1 | 0.72 | 0.55 | 0.45 |
| 58 | Ireland | IRL | European Region | 0.99 | 0.99 | 0.99 | 0.05 | 1 | 0.98 | 0.02 |
| 59 | Israel | ISR | European Region | 0.98 | 0.91 | 0.93 | 0.62 | 0.96 | 0.99 | 0.01 |
| 60 | Italy | ITA | European Region | 0.89 | 0.89 | 0.94 | 0.23 | 0.99 | 0.99 | 0.01 |
| 61 | Jamaica | JAM | Region of the Americas | 0.41 | 0.64 | 0.08 | 0.84 | 0.29 | 0.65 | 0.35 |
| 62 | Japan | JPN | Western Pacific Region | 0.99 | 0.98 | 1 | 0.02 | 0.99 | 1 | 0 |
| 63 | Jordan | JOR | Eastern Mediterranean Region | 0.81 | 0.62 | 0.32 | 0.41 | 0.37 | 0.39 | 0.61 |
| 64 | Kazakhstan | KAZ | European Region | 0.69 | 0.11 | 0.95 | 0.04 | 0.84 | 0.11 | 0.89 |
| 65 | Kenya | KEN | African Region | 0.22 | 0.05 | 0.04 | 0.97 | 0.04 | 0.01 | 0.99 |
| 66 | Korea, Rep. | KOR | Western Pacific Region | 0.99 | 0.94 | 0.97 | 0.2 | 0.96 | 0.99 | 0.01 |
| 67 | Kyrgyz Republic | KGZ | European Region | 0.26 | 0.02 | 0.73 | 0.18 | 0.04 | 0.15 | 0.85 |
| 68 | Lao PDR | LAO | Western Pacific Region | 0.09 | 0.01 | 0.04 | 0.16 | 0.07 | 0.02 | 0.98 |
| 69 | Latvia | LVA | European Region | 0.95 | 0.92 | 0.91 | 0.42 | 0.84 | 0.44 | 0.56 |
| 70 | Lebanon | LBN | Eastern Mediterranean Region | 0.88 | 0.05 | 0.61 | 0.9 | 0.65 | 0.47 | 0.53 |
| 71 | Liberia | LBR | African Region | 0.04 | 0.01 | 0.02 | 0.33 | 0.02 | 0 | 1 |
| 72 | Lithuania | LTU | European Region | 0.98 | 0.94 | 0.96 | 0.24 | 0.89 | 0.34 | 0.66 |
| 73 | Luxembourg | LUX | European Region | 0.9 | 1 | 1 | 0.02 | 1 | 0.99 | 0.01 |
| 74 | Madagascar | MDG | African Region | 0.02 | 0.1 | 0.02 | 0.89 | 0.03 | 0.02 | 0.98 |
| 75 | Malawi | MWI | African Region | 0.02 | 0.28 | 0.03 | 0.61 | 0.02 | 0 | 1 |
| 76 | Malaysia | MYS | Western Pacific Region | 0.93 | 0.83 | 0.18 | 0.87 | 0.86 | 0.48 | 0.52 |
| 77 | Mali | MLI | African Region | 0.02 | 0.14 | 0.02 | 0.44 | 0.03 | 0 | 1 |
| 78 | Malta | MLT | European Region | 0.92 | 0.98 | 0.95 | 0.03 | 0.96 | 0.98 | 0.02 |
| 79 | Mauritania | MRT | African Region | 0 | 0.03 | 0.02 | 0.54 | 0.06 | 0.01 | 0.99 |
| 80 | Mauritius | MUS | African Region | 0.59 | 0.94 | 0.39 | 0.19 | 0.71 | 0.44 | 0.56 |
| 81 | Mexico | MEX | Region of the Americas | 0.41 | 0.53 | 0.28 | 0.97 | 0.71 | 0.72 | 0.28 |
| 82 | Moldova | MDA | European Region | 0.42 | 0.25 | 0.87 | 0.15 | 0.07 | 0.22 | 0.78 |
| 83 | Mongolia | MNG | Western Pacific Region | 0.4 | 0.53 | 0.79 | 0.06 | 0.32 | 0.06 | 0.94 |
| 84 | Montenegro | MNE | European Region | 0.78 | 0.64 | 0.66 | 0.01 | 0.64 | 0.64 | 0.36 |
| 85 | Morocco | MAR | Eastern Mediterranean Region | 0.17 | 0.32 | 0.05 | 0.58 | 0.16 | 0.41 | 0.59 |
| 86 | Mozambique | MOZ | African Region | 0.01 | 0.26 | 0.02 | 0.97 | 0.02 | 0 | 1 |
| 87 | Namibia | NAM | African Region | 0.07 | 0.82 | 0.24 | 1 | 0.32 | 0.02 | 0.98 |
| 88 | Nepal | NPL | South-East Asian Region | 0.03 | 0.02 | 0.31 | 0.81 | 0.03 | 0.08 | 0.92 |
| 89 | Netherlands | NLD | European Region | 1 | 0.99 | 1 | 0.02 | 1 | 0.99 | 0.01 |
| 90 | Nicaragua | NIC | Region of the Americas | 0.07 | 0.1 | 0.05 | 0.9 | 0.08 | 0.46 | 0.54 |
| 91 | Nigeria | NGA | African Region | 0.04 | 0 | 0.03 | 0.91 | 0.1 | 0 | 1 |
| 92 | Norway | NOR | European Region | 1 | 1 | 1 | 0.01 | 1 | 0.98 | 0.02 |
| 93 | Pakistan | PAK | Eastern Mediterranean Region | 0.03 | 0 | 0.04 | 0.01 | 0.08 | 0.02 | 0.98 |
| 94 | Panama | PAN | Region of the Americas | 0.44 | 0.7 | 0.26 | 0.99 | 0.72 | 0.84 | 0.16 |
| 95 | Paraguay | PRY | Region of the Americas | 0.05 | 0.05 | 0.09 | 0.95 | 0.22 | 0.38 | 0.62 |
| 96 | Peru | PER | Region of the Americas | 0.36 | 0.39 | 0.09 | 0.96 | 0.44 | 0.55 | 0.45 |
| 97 | Philippines | PHL | Western Pacific Region | 0.57 | 0.21 | 0.13 | 0.85 | 0.13 | 0.06 | 0.94 |
| 98 | Poland | POL | European Region | 0.95 | 0.93 | 0.85 | 0.14 | 0.86 | 0.81 | 0.19 |
| 99 | Portugal | PRT | European Region | 0.95 | 0.97 | 0.89 | 0.45 | 0.94 | 0.98 | 0.02 |
| 100 | Romania | ROM | European Region | 0.75 | 0.72 | 0.84 | 0.19 | 0.78 | 0.48 | 0.52 |
| 101 | Russian Federation | RUS | European Region | 0.86 | 0.04 | 0.99 | 0.06 | 0.89 | 0.12 | 0.88 |
| 102 | Rwanda | RWA | African Region | 0.05 | 0.31 | 0.05 | 0.96 | 0.03 | 0.02 | 0.98 |
| 103 | Senegal | SEN | African Region | 0.08 | 0.44 | 0.02 | 0.63 | 0.03 | 0.03 | 0.97 |
| 104 | Serbia | SRB | European Region | 0.43 | 0.52 | 0.77 | 0.01 | 0.59 | 0.56 | 0.44 |
| 105 | Sierra Leone | SLE | African Region | 0.01 | 0.04 | 0.02 | 0.81 | 0.02 | 0 | 1 |
| 106 | Slovak Republic | SVK | European Region | 0.83 | 0.94 | 0.94 | 0.01 | 0.92 | 0.72 | 0.28 |
| 107 | Slovenia | SVN | European Region | 0.99 | 0.96 | 0.92 | 0.01 | 0.95 | 0.97 | 0.03 |
| 108 | South Africa | ZAF | African Region | 0.48 | 0.8 | 0.06 | 1 | 0.56 | 0.01 | 0.99 |
| 109 | Spain | ESP | European Region | 0.96 | 0.95 | 0.94 | 0.37 | 0.98 | 0.99 | 0.01 |
| 110 | Sri Lanka | LKA | South-East Asian Region | 0.48 | 0.29 | 0.2 | 0.27 | 0.33 | 0.47 | 0.53 |
| 111 | Suriname | SUR | Region of the Americas | 0.17 | 0.56 | 0.48 | 0.97 | 0.64 | 0.18 | 0.82 |
| 112 | Swaziland | SWZ | African Region | 0.04 | 0.08 | 0.14 | 0.99 | 0.23 | 0 | 1 |
| 113 | Sweden | SWE | European Region | 1 | 1 | 0.99 | 0.01 | 1 | 0.99 | 0.01 |
| 114 | Switzerland | CHE | European Region | 1 | 1 | 1 | 0.03 | 1 | 0.99 | 0.01 |
| 115 | Tajikistan | TJK | European Region | 0.16 | 0 | 0.74 | 0.05 | 0.03 | 0.09 | 0.91 |
| 116 | Tanzania | TZA | African Region | 0.01 | 0.23 | 0.03 | 0.42 | 0.03 | 0 | 1 |
| 117 | Thailand | THA | South-East Asian Region | 0.75 | 0.41 | 0.1 | 0.96 | 0.62 | 0.47 | 0.53 |
| 118 | Timor-Leste | TMP | South-East Asian Region | 0.02 | 0.02 | 0.45 | 0.18 | 0.03 | 0.05 | 0.95 |
| 119 | Trinidad and Tobago | TTO | Region of the Americas | 0.49 | 0.72 | 0.42 | 0.54 | 0.97 | 0.16 | 0.84 |
| 120 | Tunisia | TUN | Eastern Mediterranean Region | 0.87 | 0.53 | 0.19 | 0.45 | 0.46 | 0.52 | 0.48 |
| 121 | Turkey | TUR | European Region | 0.57 | 0.6 | 0.3 | 0.7 | 0.81 | 0.59 | 0.41 |
| 122 | Uganda | UGA | African Region | 0.02 | 0.09 | 0.96 | 0.84 | 0.03 | 0 | 1 |
| 123 | Ukraine | UKR | European Region | 0.87 | 0.09 | 0.74 | 0.01 | 0.27 | 0.16 | 0.84 |
| 124 | United Kingdom | GBR | European Region | 0.99 | 0.99 | 0.96 | 0.2 | 0.99 | 0.98 | 0.02 |
| 125 | United States | USA | Region of the Americas | 1 | 0.98 | 1 | 0.91 | 1 | 0.93 | 0.07 |
| 126 | Uruguay | URY | Region of the Americas | 0.8 | 0.94 | 1 | 0.8 | 0.74 | 0.75 | 0.25 |
| 127 | Venezuela | VEN | Region of the Americas | 0.46 | 0 | 0.17 | 0.98 | 0.74 | 0.39 | 0.61 |
| 128 | Vietnam | VNM | Western Pacific Region | 0.18 | 0.13 | 0.03 | 0.06 | 0.08 | 0.62 | 0.38 |
| 129 | Yemen | YEM | Eastern Mediterranean Region | 0.01 | 0 | 0.36 | 0.19 | 0.07 | 0.02 | 0.98 |
| 130 | Zambia | ZMB | African Region | 0.06 | 0.32 | 0.08 | 0.96 | 0.05 | 0 | 1 |
| 131 | Zimbabwe | ZWE | African Region | 0.06 | 0 | 0.07 | 0.97 | 0.03 | 0 | 1 |
