# Supplementary material for: A fuzzy set qualitative comparative analysis of 131 countries: which configuration of the structural conditions can explain health better?
Source: Int J Equity Health. 2018 Jan 22;17:10. doi: 10.1186/s12939-018-0724-1 (PMC5778742; doi:10.1186/s12939-018-0724-1)
Supplement: Supplementary file 4 — fsQCA Complex and Parsimonious solutions. (DOCX 40 kb) [file 12939_2018_724_MOESM4_ESM.docx]

Additional file 4 **fsQCA Complex and parsimonious solutions**

Table 1) fsQCA solutions for high life expectancy (frequency cutoff: 2, consistency cutoff: 0.9)

| Configuration | Raw coverage | Unique coverage | Consistency |
| --- | --- | --- | --- |
| **Complex solution,** | | | |
| E* G * H * W | 0.789 | 0.789 | 0.946 |
| solution coverage: 0.789, solution consistency: 0.946 |  |  |  |
| **Parsimonious solution** | | | |
| G * H | 0.808 | 0.808 | 0.907 |
| solution coverage: 0.808, solution consistency: 0.907 | | | |
| **Intermediate solution** | | | |
| E* G * H * W | 0.789 | 0.789 | 0.946 |
| solution coverage: 0.789, solution consistency: 0.946 | | | |
| ‘*’ means AND, ‘+’ means OR Upper case: high level (>0.5); Lower case: low level (<0.5). | | | |

Table 2) fsQCA solutions for low life expectancy using original calibration (frequency cutoff: 2, consistency cutoff: 0.9)

| Configuration | Raw coverage | Unique coverage | consistency |
| --- | --- | --- | --- |
| **Complex solution** | | | |
| (e * h * I) + | 0.539 | 0.302 | 0.953 |
| (e *g * i* w) + | 0.331 | 0.134 | 0.995 |
| (g * h* I * W) + | 0.154 | 0.013 | 0.961 |
| (E *g * H* i* W) | 0.113 | 0.024 | 0.946 |
| solution coverage: 0.745, solution consistency: 0.955 | | | |
| **Parsimonious solution** | | | |
| e + | 0.846 | 0.149 | 0.947 |
| (g * i) + | 0.401 | 0.035 | 0.957 |
| ( g * h) | 0.679 | 0.024 | 0.95 |
| solution coverage: 0.92, solution consistency: 0.930 | | | |
| **Intermediate solution** | | | |
| (g * h) + | 0.676 | 0.028 | 0.950 |
| (g * i) + | 0.401 | 0.045 | 0.957 |
| (e * g * w) + | 0.679 | 0.019 | 0.971 |
| (e * h * I )+ | 0.539 | 0.073 | 0.953 |
| solution coverage: 0.863, solution consistency: 0.938 | | | |
