# Supplementary material for: A fuzzy set qualitative comparative analysis of 131 countries: which configuration of the structural conditions can explain health better?
Source: Int J Equity Health. 2018 Jan 22;17:10. doi: 10.1186/s12939-018-0724-1 (PMC5778742; doi:10.1186/s12939-018-0724-1)
Supplement: Supplementary file 5 — Sensitivity analysis. (DOCX 71 kb) [file 12939_2018_724_MOESM5_ESM.docx]

Additional file 6

Sensitivity analysis

[1 Sensitivity analysis for the frequency threshold 1](#_Toc499323511)

[2 Sensitivity analysis for the crossover point in calibration 2](#_Toc499323512)

[2.1 Alternative calibration (I) 2](#_Toc499323513)

[2.2 Alternative calibration (II) 4](#_Toc499323514)

[3 Sensitivity analysis for the consistency threshold 6](#_Toc499323515)

# Sensitivity analysis for the frequency threshold

Table 1) fsQCA solutions for high life expectancy, original calibration (frequency cutoff: 1, consistency cutoff: 0.9)

| Configuration | Raw coverage | Unique coverage | Consistency |  |
| --- | --- | --- | --- | --- |
| **Complex solution,** | | | | |
| E* G * H * W | 0.789 | 0.789 | 0.946 |  |
| solution coverage: 0.789, solution consistency: 0.946 |  |  |  |  |
| **Parsimonious solution** | | | | |
| E * G * H | 0.797 | 0.797 | 0.937 |  |
| solution coverage: 0. 797, solution consistency: 0.937 | | | | |
| **Intermediate solution** | | | | |
| E* G * H * W | 0.789 | 0.789 | 0.946 |  |
| solution coverage: 0.789, solution consistency: 0.946 | | | | |
| ‘*’ means AND, ‘+’ means OR E: education; G: governance; H: health system; I: income inequality; W: wealth  Upper case: high level (>0.5); Lower case: low level (<0.5). | | | | |
|  |  |  |  |  |

Table 2) fsQCA solutions for low life expectancy, original calibration (frequency cutoff: 1, consistency cutoff: 0.9)

| Configuration | Raw coverage | Unique coverage | consistency |
| --- | --- | --- | --- |
| **Complex solution** | | | |
| (g *H * i) + | 0.194 | 0.031 | 0.944 |
| (e* H * i) + | 0.168 | 0.001 | 0.937 |
| (g* h* I) + | 0.492 | 0.006 | 0.945 |
| (e * h* I) + | 0.539 | 0.066 | 0.953 |
| (E* G * h * i) + | 0.112 | 0.009 | 0.928 |
| (g* h* w) + | 0.64 | 0.132 | 0.967 |
| (e * H * w) | 0.193 | 0.015 | 0.964 |
| solution coverage: 0.855, solution consistency: 0.930 | | | |
| **Parsimonious solution** | | | |
| e + | 0.846 | 0.139 | 0.947 |
| (h * i) + | 0.333 | 0.015 | 0.965 |
| (g * i) + | 0.401 | 0.035 | 0.957 |
| (g * h) | 0.676 | 0.024 | 0.95 |
| solution coverage: 0.935, solution consistency: 0.926 | | | |
| **Intermediate solution** | | | |
| e + | 0.846 | 0.139 | 0.947 |
| (g * h) + | 0.676 | 0.024 | 0.95 |
| (g * i) + | 0.401 | 0.035 | 0.957 |
| (h * i ) | 0.333 | 0.015 | 0.965 |
| solution coverage: 0.935, solution consistency: 0.926 | | | |
|  | | |  |

# Sensitivity analysis for the crossover point in calibration

## Alternative calibration (I)

The cross over point was set at 40^th^ percentile of the raw data in the new calibrations of each condition.

Table 3) Threshold values for alternative calibration (I)

| Condition set | Fully in | cross-over point | Fully out |
| --- | --- | --- | --- |
|  |  | **≈** 40^th^ Percentile |  |
| High Life expectancy | 80 | 72.5 | 68 |
| Low Life expectancy | 68 | 72.5 | 80 |
| High education (E) | 4.86 | 3.7 | 3.04 |
| Good Governance (G) | 0.86 | -0.32 | -0.69 |
| Affluent health system (H) | 0.9 | -0.43 | -0.7 |
| High income inequality (I) | 33 | 18.5 | 15 |
| High Income (W) | 28500 | 7800 | 3000 |
|  |  |  |  |

No individual conditions were identified to be necessary for high or low life expectancy.

Table 4) fsQCA solutions for high life expectancy, alternative calibration (I) **(**consistency cutoff: 0.9)

| Configuration | Raw coverage | Unique coverage | consistency |
| --- | --- | --- | --- |
| **Complex solution, frequency cutoff: 1 & 2** | | | |
| E * G * H * i * W | 0.579 | 0. 579 | 0.965 |
| solution coverage: 0.579, solution consistency: 0.965 | | | |
| **Parsimonious solution, frequency cutoff: 1** | | | |
| G * i * W | 0.592 | 0.592 | 0.944 |
| solution coverage: 0.592, solution consistency: 0.944 | | | |
| **Parsimonious solution, frequency cutoff: 2** | | | |
| G * i | 0.6 | 0. 6 | 0.88 |
| solution coverage: 0.6, solution consistency: 0.88 | | | |
| **Intermediate solution, frequency cutoff: 1 & 2** | | | |
| E * G * H * i * W | 0.579 | 0. 579 | 0.965 |
| solution coverage: 0.579, solution consistency: 0.965 | | | |

Table 5) fsQCA solutions for low life expectancy, alternative calibration (I) (consistency cutoff: 0.9)

| Configuration | Raw coverage | | Unique coverage | consistency |
| --- | --- | --- | --- | --- |
| **Complex solution, frequency cutoff: 1** | | | | |
| (e * g * w )+ | | 0.629 | 0.093 | 0.944 |
| (g * H * w) + | | 0.256 | 0.02 | 0.934 |
| (e * g * H) + | | 0.231 | 0.004 | 0.977 |
| (g * I * w) + | | 0.526 | 0.023 | 0.933 |
| (e* g* I) + | | 0.507 | 0.01 | 0.929 |
| (e * h * I * w) + | | 0.508 | 0.051 | 0.942 |
| (e* H* I * W) + | | 0.142 | 0.008 | 0.966 |
| E* G * h * i * w) + | | 0.103 | 0.007 | 0.955 |
| E * g * h * i * W) | | 0.104 | 0.004 | 0.953 |
| solution coverage: 0.813, solution consistency: 0.917 | | | | |
| **Complex solution, frequency cutoff: 2** | |  |  |  |
| (e * g * w) + | | 0.629 | 0.123 | 0.944 |
| (g * H * i * w) + | | 0.16 | 0.022 | 0.925 |
| (g * h * I * w) + | | 0.482 | 0.032 | 0.931 |
| (e * h * I * w) + | | 0.508 | 0.052 | 0.942 |
| (e * G* H * I * W) | | 0.113 | 0.015 | 0.958 |
| solution coverage: 0.769, solution consistency: 0.927 | | | |  |
| **Parsimonious solution, frequency cutoff: : 1** | | | | |
| e + | 0.785 | | 0.144 | 0.93 |
| (h * i) + | 0.284 | | 0.02 | 0.966 |
| (g * w) | 0.706 | | 0.061 | 0.923 |
| solution coverage: 0.884, solution consistency: 0.912 | | | | |
| **Parsimonious solution, frequency cutoff: : 2** | | | | |
| w + | 0.832 | | 0.108 | 0.899 |
| e | 0.785 | | 0.061 | 0.93 |
| solution coverage: 0.894, solution consistency: 0.891 | | | |  |
| **Intermediate solution, frequency cutoff: : 1** | | | | |
| (e * g) + | 0.667 | | 0.012 | 0.931 |
| (g * w) + | 0.706 | | 0.061 | 0.923 |
| (e * I) + | 0.595 | | 0.084 | 0.929 |
| (g * h * i) + | 0.265 | | 0.004 | 0.98 |
| (h * i * w) | 0.267 | | 0.01 | 0.982 |
| solution coverage: 0.849, solution consistency: 0.914 | | | |  |
| **Intermediate solution, frequency cutoff: : 2** |  | |  |  |
| (g * w) + | 0.706 | | 0.224 | 0.923 |
| (e * I) + | 0.595 | | 0.112 | 0.929 |
| solution coverage: 0.819, solution consistency: 0.914 | | | | |
|  | | | | |

## Alternative calibration (II)

The cross over point was set at 60^th^ percentile of the raw data in the new calibrations of each condition.

Table 6) Threshold values for alternative calibration (II)

| Interval-scale | Fully in | cross-over point **≈** 60^th^ Percentile | Fully out |
| --- | --- | --- | --- |
| High Life expectancy | 80 | 76.4 | 68 |
| Low Life expectancy | 68 | 76.4 | 80 |
| High education | 4.86 | 4.26 | 3.04 |
| Good Governance | 0.86 | 0.04 | -0.69 |
| Affluent health system | 0.9 | 0.11 | -0.7 |
| High Income | 28500 | 14300 | 3000 |
| High income inequality | 33 | 24 | 15 |

No individual conditions were found to be necessary for high or low level of life expectancy.

Table 7) fsQCA solution for high life expectancy, alternative calibration (II), (consistency cutoff: 0.9)

| Configuration | Raw coverage | Unique coverage | consistency |
| --- | --- | --- | --- |
| **Complex solution, frequency cutoff: 1 & 2** | | | |
| E * G * H * i *W | 0.721 | 0.721 | 0.946 |
| solution coverage: 0.721, solution consistency: 0.946 | | | |
| **Parsimonious solution, frequency cutoff: 1 & 2** | | | |
| G * H * i | 0.734 | 0.734 | 0.921 |
| solution coverage: 0.734, solution consistency: 0.921 | | | |
| **Intermediate solution, frequency cutoff: 1 & 2** | | | |
| E * G * H * i *W | 0.721 | 0.721 | 0.946 |
| solution coverage: 0.721, solution consistency: 0.946 | | | |

Table 8) fsQCA solutions for low life expectancy, alternative calibration (II) (consistency cutoff: 0.9)

| Configuration | Raw coverage | Unique coverage | consistency |
| --- | --- | --- | --- |
| **Complex solution, frequency cutoff: 1** | | | |
| ( g * w) + | 0.778 | 0.075 | 0.966 |
| (e * h * w) + | 0.634 | 0.013 | 0.982 |
| (h * I * w) + | 0.484 | 0.013 | 0.948 |
| (E * g * H) + | 0.183 | 0.021 | 0.944 |
| (E * g * I) + | 0.195 | 0.012 | 0.905 |
| ( e * g * h * i) | 0.318 | 0.004 | 0.992 |
| solution coverage: 0.88, solution consistency: 0.938 |  |  |  |
| **Complex solution, frequency cutoff: 2** |  |  |  |
| ( g * h* w) + | 0.699 | 0.219 | 0.970 |
| (h * I * w) + | 0.484 | 0.043 | 0.948 |
| (E * g *H * i) + | 0.154 | 0.061 | 0.94 |
| (E* g * h * I) | 0.189 | 0.013 | 0.953 |
| solution coverage: 0.821, solution consistency: 0.947 |  |  |  |
| **Parsimonious solution, frequency cutoff: 1 & 2** | | | |
| w + | 0.868 | 0.09 | 0.946 |
| g | 0.842 | 0.064 | 0.929 |
| solution coverage: 0.933, solution consistency: 0.915 | | | |
| **Intermediate solution, frequency cutoff: 1** | | | |
| g + | 0.842 | 0.192 | 0.929 |
| e * h * w | 0.634 | 0.013 | 0.982 |
| h * I * w | 0.484 | 0.013 | 0.948 |
| solution coverage: 0.899, solution consistency: 0.922 |  |  |  |
| **Intermediate solution, frequency cutoff: 2** |  |  |  |
| g + | 0.842 | 0.401 | 0.929 |
| h * I * w | 0.484 | 0.043 | 0.948 |
| solution coverage: 0.885, solution consistency: 0.921 |  |  |  |

#

# Sensitivity analysis for the consistency threshold

Table 9) fsQCA solutions for high life expectancy, original calibration (consistency cutoff: 0.84)

| Configuration | Raw coverage | Unique coverage | consistency |
| --- | --- | --- | --- |
| **Complex solution, frequency cutoff: 1 & 2** | | | |
| (E * G * H * W) + | 0.789 | 0.586 | 0.946 |
| (E * H * I * W) | 0.224 | 0.021 | 0.893 |
| solution coverage: 0.811, solution consistency: 0.94 | | | |
| **Parsimonious solution,** **frequency cutoff: 1** | | | |
| (E * G *H) + | 0.797 | 0.59 | 0.937 |
| (E * H * I) | 0.23 | 0.024 | 0.885 |
| solution coverage: 0.821, solution consistency: 0.93 |  |  |  |
| **Parsimonious solution,** **frequency cutoff: 2** |  |  |  |
| (H * I) + | 0.237 | 0.027 | 0.729 |
| (G * H) | 0.808 | 0.598 | 0.907 |
| solution coverage: 0.836, solution consistency: 0.867 | | | |
| **Intermediate solution, frequency cutoff: 1 & 2** | | | |
| (E * G * H * W) + | 0.789 | 0.586 | 0.946 |
| (E * H * I * W) | 0.224 | 0.021 | 0.893 |
| solution coverage: 0.811, solution consistency: 0.94 | | | |
|  | | | |

Table 10) fsQCA solutions for low life expectancy, original calibration (consistency cutoff: 0.94)

| Configuration | Raw coverage | Unique coverage | consistency |
| --- | --- | --- | --- |
| **Complex solution, frequency cutoff: 1** | | | |
| (g * H* i ) + | 0.194 | 0.030 | 0.944 |
| (e* H * w) + | 0.193 | 0.008 | 0.964 |
| (g * h * I ) + | 0.492 | 0.006 | 0.945 |
| (e* h * I ) + | 0.539 | 0.068 | 0.953 |
| (E* h * I * w) + | 0.124 | 0.006 | 0.982 |
| (g * h* w) + | 0.64 | 0.003 | 0.967 |
| (e * g * W ) | 0.679 | 0.015 | 0.971 |
| solution coverage: 0.846, solution consistency: 0.939 | | | |
| **Complex solution, frequency cutoff: 2** | |  |  |
| ( e * h * I ) + | 0.539 | 0.302 | 0.953 |
| ( e * g * i * w) + | 0.331 | 0.134 | 0.995 |
| (g * h *I * W) + | 0.154 | 0.013 | 0.961 |
| ( E * g * H * i * W) | 0.113 | 0.024 | 0.946 |
| solution coverage: 0.745, solution consistency: 0.955 | | | |
| **Parsimonious solution, frequency cutoff: 1** | | | |
| w + | 0.839 | 0.052 | 0.944 |
| (g * i) + | 0.401 | 0.033 | 0.957 |
| ( g * h ) + | 0.676 | 0.007 | 0.95 |
| (e * h) | 0.741 | 0.017 | 0.955 |
| solution coverage: 0.926, solution consistency: 0.922 | | |  |
| **Parsimonious solution, frequency cutoff: 2** | | | |
| **e +** | 0.846 | 0.149 | 0.947 |
| **(g * i )+** | 0.401 | 0.035 | 0.957 |
| (g * h) | 0.676 | 0.024 | 0.95 |
| solution coverage: 0.92, solution consistency: 0.93 | | | |
| **Intermediate solution,** **frequency cutoff: 1** | | | |
| **(g *** h**) +** | 0.676 | 0.011 | 0.95 |
| **(**g * i **)+** | 0.401 | 0.045 | 0.957 |
| (e * w) + | 0.774 | 0.028 | 0.962 |
| ( h * w) + | 0.743 | 0.017 | 0.954 |
| (e * h * I) | 0.539 | 0.015 | 0.953 |
| solution coverage: 0.917, solution consistency: 0.928 | | | |
| **Intermediate solution,** **frequency cutoff: 2** | | | |
| (g * h) + | 0.676 | 0.028 | 0.95 |
| (g * i ) + | 0.401 | 0.045 | 0.957 |
| ( e * g * w) + | 0.679 | 0.019 | 0.971 |
| (e * h * I) | 0.539 | 0.073 | 0.953 |
| solution coverage: 0.863, solution consistency: 0.938 | | | |
