# Supplementary material for: A fuzzy set qualitative comparative analysis of 131 countries: which configuration of the structural conditions can explain health better?
Source: Int J Equity Health. 2018 Jan 22;17:10. doi: 10.1186/s12939-018-0724-1 (PMC5778742; doi:10.1186/s12939-018-0724-1)
Supplement: Supplementary file 6 — Distribution of cases across combinations of causal conditions (Contradictory cases are shown in bold text). (DOCX 22 kb) [file 12939_2018_724_MOESM6_ESM.docx]

Additional file 5 Distribution of cases across combinations of causal conditions **(**Bold text indicates contradictory cases)

|  | |  |  |  |  |  |  | Consistency for | |  |
| --- | --- | --- | --- | --- | --- | --- | --- | --- | --- | --- |
|  | | Education | Governance | Health System | Income Inequality | wealth | number | high life expectancy | low life expectancy | Cases |
|  | 1 | | 1 | 1 | 0 | 1 | 36 | 0.929 | 0.285 | Australia Austria Barbados Belgium **Bulgaria** Canada Cyprus Czech Republic Denmark Estonia Finland France Germany Greece Hungary Iceland Ireland Italy Japan Korea, Rep. **Latvia** **Lithuania** Luxembourg Malta Montenegro Netherlands Norway Poland Portugal **Romania** Slovak Republic Slovenia Spain Sweden Switzerland United Kingdom |
|  | 1 | | 1 | 1 | 1 | 1 | 5 | 0.925 | 0.649 | **Brazil** Croatia Israel Uruguay United States |
|  | 1 | | 0 | 1 | 1 | 1 | 2 | 0.889 | 0.894 | Argentina Lebanon |
|  | 1 | | 0 | 0 | 1 | 1 | 2 | 0.873 | 0.945 | Colombia Thailand |
|  | 1 | | 1 | 0 | 1 | 1 | 4 | 0.845 | 0.806 | Chile Costa Rica Malaysia Turkey |
|  | 1 | | 0 | 0 | 1 | 0 | 2 | 0.808 | 0.96 | **China** Philippines |
|  | 1 | | 1 | 0 | 0 | 0 | 2 | 0.807 | 0.952 | Jordan **Tunisia** |
|  | 0 | | 0 | 0 | 1 | 1 | 4 | 0.765 | 0.934 | Dominican Republic Gabon **Iran**  Venezuela |
|  | 1 | | 0 | 1 | 0 | 1 | 2 | 0.752 | 0.934 | Kazakhstan Russian Federation |
|  | 0 | | 1 | 0 | 1 | 1 | 6 | 0.733 | 0.912 | Botswana **Mexico** **Panama** Suriname Trinidad and Tobago South Africa |
|  | 0 | | 0 | 1 | 1 | 0 | 2 | 0.703 | 0.947 | **Bosnia and Herzegovina** Uganda |
|  | 0 | | 0 | 1 | 0 | 0 | 4 | 0.616 | 0.953 | Armenia Kyrgyz Republic Moldova Tajikistan |
|  | 0 | | 1 | 0 | 1 | 0 | 7 | 0.537 | 0.953 | Belize Bhutan Cape Verde El Salvador Ghana **Jamaica** Namibia |
|  | 0 | | 0 | 0 | 0 | 0 | 16 | 0.36 | 0.949 | **Albania** Burundi Cambodia Cameroon Egypt Ethiopia India Lao PDR Liberia Mali Pakistan Sri Lanka Tanzania Timor-Leste **Vietnam** Yemen |
|  | 0 | | 0 | 0 | 1 | 0 | 30 | 0.332 | 0.95 | Angola Bangladesh Benin Bolivia Burkina Faso Chad Côte d'Ivoire **Ecuador** Gambia, The Guatemala Guinea Guyana Honduras Kenya Madagascar Malawi Mauritania Morocco Mozambique Nepal Nicaragua Nigeria Paraguay **Peru** Rwanda Senegal Sierra Leone Swaziland Zambia Zimbabwe |
